# Mechanisms of Dietary Response in Mice and Primates: A Role for EGR1 in Regulating the Reaction to Human-Specific Nutritional Content

**DOI:** 10.1371/journal.pone.0043915

**Published:** 2012-08-24

**Authors:** Kai Weng, Haiyang Hu, Augix Guohua Xu, Philipp Khaitovich, Mehmet Somel

**Affiliations:** 1 Key Laboratory of Computational Biology, CAS-MPG Partner Institute for Computational Biology, Chinese Academy of Sciences, Shanghai, China; 2 Max Planck Institute for Evolutionary Anthropology, Leipzig, Germany; 3 Department of Integrative Biology, University of California, Berkeley, California, United States of America; Klinikum rechts der Isar der TU München, Germany

## Abstract

**Background:**

Humans have a widely different diet from other primate species, and are dependent on its high nutritional content. The molecular mechanisms responsible for adaptation to the human diet are currently unknown. Here, we addressed this question by investigating whether the gene expression response observed in mice fed human and chimpanzee diets involves the same regulatory mechanisms as expression differences between humans and chimpanzees.

**Results:**

Using mouse and primate transcriptomic data, we identified the transcription factor EGR1 (early growth response 1) as a putative regulator of diet-related differential gene expression between human and chimpanzee livers. Specifically, we predict that EGR1 regulates the response to the high caloric content of human diets. However, we also show that close to 90% of the dietary response to the primate diet found in mice, is not observed in primates. This might be explained by changes in tissue-specific gene expression between taxa.

**Conclusion:**

Our results suggest that the gene expression response to the nutritionally rich human diet is partially mediated by the transcription factor EGR1. While this EGR1-driven response is conserved between mice and primates, the bulk of the mouse response to human and chimpanzee dietary differences is not observed in primates. This result highlights the rapid evolution of diet-related expression regulation and underscores potential limitations of mouse models in dietary studies.

## Introduction

Dietary change has been proposed as one of the main driving forces of human evolution, as well as one of the major causes of modern-day common metabolic disorders (reviewed in [1]–[4]). In the past several million years, the human diet has undergone several major shifts, including reliance on tubers, increased consumption of meat, the invention of food processing methods like cooking, and, more recently, a switch to high-calorie diets based on domesticated crops and animals. These changes may underlie multiple evolutionary adaptations [5]. For example, it has been suggested that switching to high-quality processed food has resulted in reduced tooth and gut size during human evolution, as well as greater encephalization [6]–[8]. The effects of dietary change in human evolution can be further detected among current-day populations with different traditional diets. A classic example is the lactase persistence phenotype present at high frequencies in populations with a long history of milk consumption [9]–[11]. Moreover, common metabolic disorders such as obesity and diabetes might be explained by recent changes in dietary trends incompatible with earlier dietary adaptations [4], [12].

Regardless of local differences, all known human societies share common dietary features that contrast with the fruit- and leaf-based chimpanzee diets [13]. These features include higher protein content, more calories, and cooking. Importantly, all these features are critical for health in contemporary humans [14], [15], indicating the presence of irreversible genetic adaptations to these new dietary conditions. Despite the suggested relevance of the dietary changes to human evolution, diet-associated genetic adaptations common to all humans are not yet known. Furthermore, the general impact of dietary differences on human and chimpanzee phenotypic differences is not well understood [16]. A major obstacle here is the lack of a consensus model for studying the physiological and molecular effects of the dietary shifts observed during human evolution.

Mouse models have been widely employed to study metabolic disorders such as type 2 diabetes [17] and non-alcoholic fatty liver disease [18], and are therefore good candidates for investigating the effects of dietary change in human evolution. Accordingly, a recent study used mice to analyze differential effects of human and chimpanzee diets on gene expression [19]. The results showed that within two weeks, differences between human and chimpanzee diets resulted in conspicuous expression changes in the mouse liver, but not in the brain. Furthermore, genes differentially expressed due to diet differences in mouse liver also tended to be differentially expressed between human and chimpanzee livers, suggesting a role for diet differences in the two species' transcriptome differences. However, the regulatory mechanisms driving these diet-related expression patterns in rodents and in primates were not examined. Likewise, the study did not address the causes of expression differences detected only in mice fed human and chimpanzee diets, but not detected in humans and chimpanzees. Here we address these issues by investigating shared and divergent gene expression regulation between mice and primates with respect to dietary response, with a specific focus on *trans* regulation by transcription factors.

## Results

### Transcription factors regulating human vs. chimpanzee differences in liver

**Figure 1:**
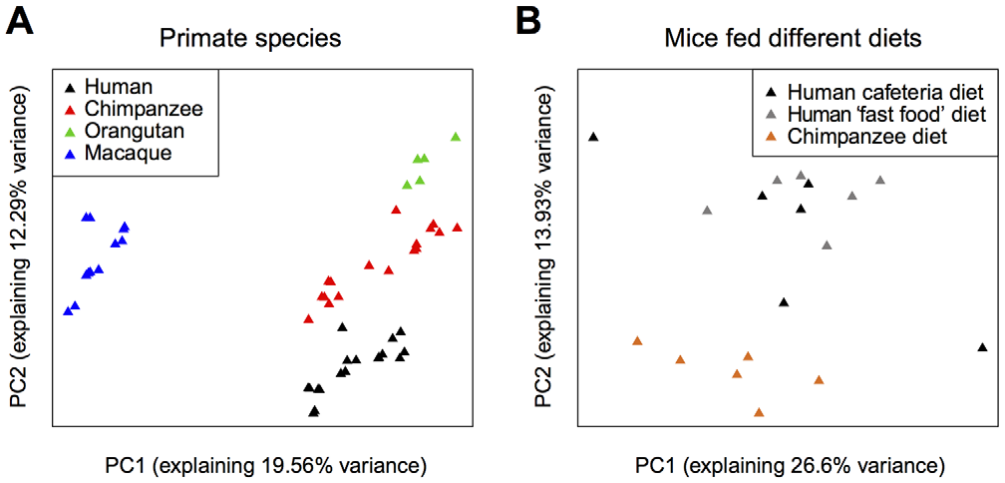
Liver gene expression variance among primate species and mice fed human and chimpanzee diets. The first two principal components of liver gene expression (A) in four primate species (the combined primate dataset, including the RNA-sequencing and microarrays datasets) and (B) in mice fed human ‘cafeteria’, human ‘fast-food’, or chimpanzee diets. The analysis was performed by singular value decomposition, using the “prcomp” function in the R “stats” package [60]); each gene's expression level was scaled to unit variance before analysis, to yield a z-score. The proportion of variance explained by each principal component is shown in parentheses.

We quantified gene expression in the livers of humans, chimpanzees, and outgroup species using two independent public datasets (Methods). The first dataset was based on microarray measurements from six humans, five chimpanzees, and five orangutans [20], [21]. Among 4,531 reliably detected genes, 969 (21%) were significantly differentially expressed between humans and chimpanzees (permutation-based false discovery rate [FDR] <10%). The second dataset was based on RNA-sequencing (RNA-seq) measurements from 12 humans, 12 chimpanzees and 12 macaques [22]. Among 13,145 genes reliably detected in this latter dataset, 4,551 (35%) were significantly differentially expressed between human and chimpanzee livers (permutation-based FDR <10%). Both detected and differentially expressed genes showed significant overlap between the two datasets (4,161 and 446 genes, odds ratio  = 13.31 and 3.01, one-sided Fisher's exact test, *p*<1×10^−10^, respectively). The amplitude and direction of expression differences between human and chimpanzee livers also showed good agreement between these two datasets (Pearson correlation, *r* = 0.82, *p*<1×10^−10^; Figure S1). We, therefore, combined the two datasets based on 4,161 commonly detected genes for further analyses. Principal components analysis (PCA) of this combined dataset demonstrated a clear separation among samples according to their species identity (Figure 1A), indicating a large impact of species differences on total transcriptome variation. In agreement with this observation, using an absolute effect size cutoff (|effect size|>0.8) corresponding to a cumulative two-dataset FDR <5%, 1,792 genes (43.1%) showed consistent differential expression between humans and chimpanzees in the two datasets (Methods).

Previous analyses of genes differentially expressed between human and chimpanzee livers have reported enrichment in functions including metabolic functions and transcriptional regulation [22], [23]. Regulatory mechanisms responsible for these human-chimpanzee expression differences, however, have not been studied. Generally, gene expression differences between species may be caused by: (i) DNA sequence differences in *cis-*regulatory regions such as promoters and enhancers, which are hard-wired [24], and (ii) differences in the concentration or activity of *trans-*acting factors such as transcription factors (TF), microRNAs, DNA methyltransferases, or chromatin modifiers, which may or may not be hard-wired between species. More specifically, *trans* regulation could itself be the result of evolutionary *cis* changes (e.g. substitutions in a TF's promoter that constitutively up-regulate its expression), or may represent plastic responses to environmental differences between species (e.g. activation of a TF upon agonist binding) [25].

To test whether differences in TF expression might be linked to gene expression differences between human and chimpanzee livers, we examined correlations between each TF's expression and the expression of its predicted target genes. These potential target genes were defined based on the presence of conserved binding motifs, for a given TF, in their promoter region (±2,000 bp from the transcription start site [TSS]; Methods) [26]. Here we assume that a change in a TF's mRNA concentration indicates changing activity, which should be reflected in the expression levels of its targets. Thus, a TF regulating its predicted targets should show either more positive or more negative correlations with these targets, compared to its correlations with other genes, which can be evaluated by the Wilcoxon test (Methods) [27]. The null expectation, i.e. a TF showing correlation with randomly selected targets, was gauged by a permutation test.

Among 62 TFs expressed in the combined primate liver dataset, and having annotated target genes, 23 showed expression differences between humans and chimpanzees at |effect size|>0.8. These had in total 981 potential targets also showing a human-chimpanzee |effect size|>0.8. Asking if any of these TF might show non-random correlations with their targets' expression, we identified two (*EGR1* and *MEF2A*) showing more positive correlations with their own target genes than other TFs' targets genes (two-sided Wilcoxon test, *p*<0.01, Table S1). Finding two such TFs is unexpected, as estimated by permuting TF-target relationships (*p* = 0.018; Methods). Thus, differential expression of these two TFs may drive differential expression of their target genes between human and chimpanzee livers.

**Figure 2:**
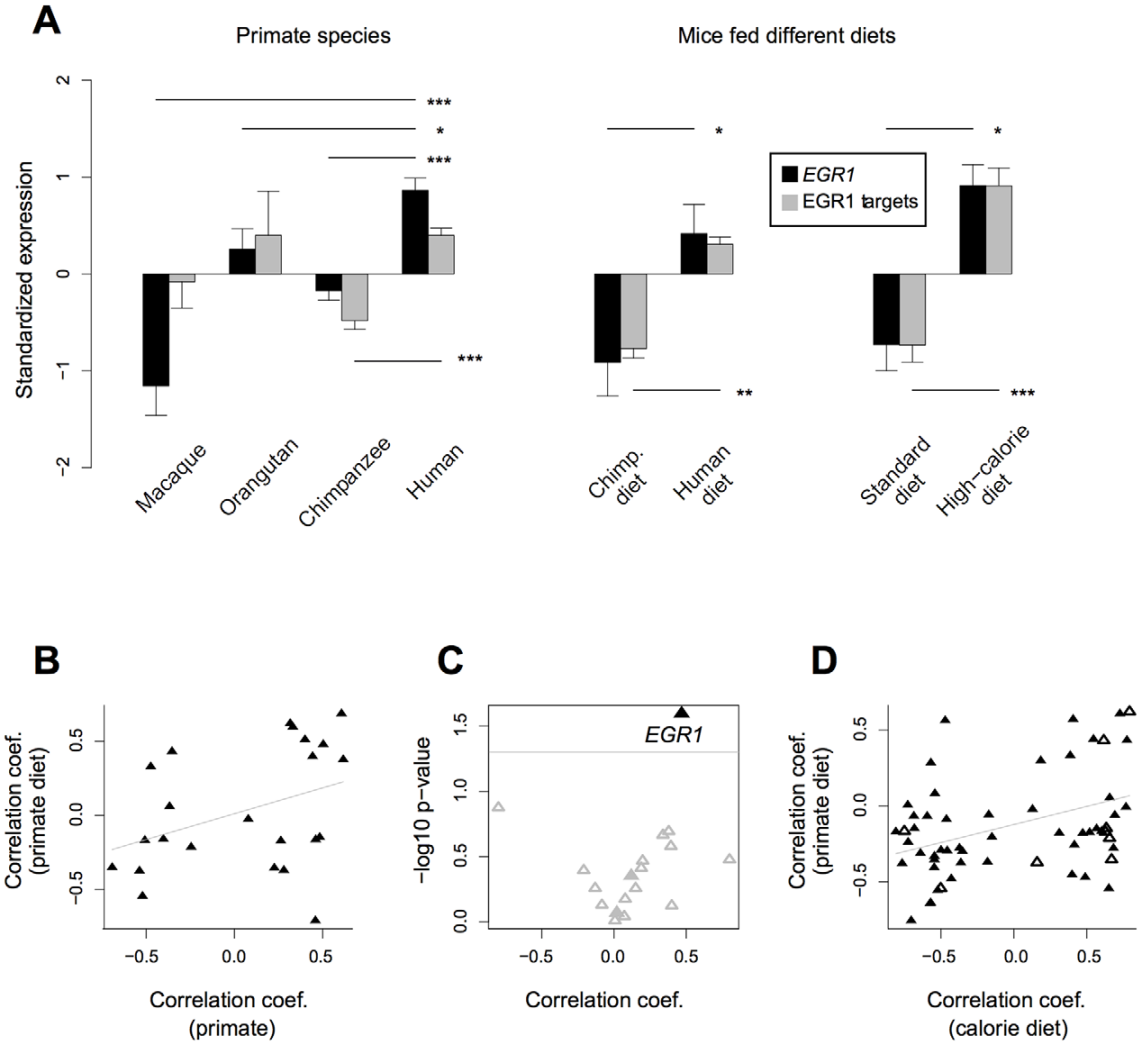
*EGR1'*s response to human-chimpanzee dietary differences. (A) Standard normalized expression of *EGR1* (black) and its target genes (gray) showing species/diet effects (|effect size|>0.8) and positive correlation with the *EGR1* expression profile (N = 7 genes in the primate dataset and the mouse primate diet dataset, and N = 10 in the high calorie diet dataset). The seven target genes in the primate dataset and the mouse primate diet dataset were chosen based on positive correlation with *EGR1* in both datasets, while the 10 target genes in the mouse high-calorie diet dataset were chosen based on positive correlation with *EGR1* in this dataset as well as in the mouse primate diet dataset. Results are expressed as mean ± SEM for *EGR1* and mean ± SEM of mean expressions for target genes. Asterisks indicate significance based on two-sided t-tests, ***: *p*<0.001; **: *p*<0.01, *: *p*<0.05. The seven targets in the primate dataset show similar trends as *EGR1* but significant expression difference only between humans and chimpanzees. (B) Scatter plot of correlation coefficients between *EGR1* and its 23 tested targets, showing species effects in the primate dataset and diet effects in the mouse human-chimpanzee diet dataset. The x- and y-axes show the correlation coefficient between *EGR1* and each target in the primate dataset and the mouse human-chimpanzee diet dataset, respectively. The Spearman correlation coefficient between these coefficients (correlation of correlations, CoC) was 0.46 (*p* = 0.028). (C) Volcano plot of CoCs for 18 TFs tested in either the primate dataset or the mouse human-chimpanzee diet dataset (i.e. showing species/diet effects, |effect size|>0.8, in both datasets). The x-axis is the CoC between each TF and its targets shared by the two datasets. The y-axis is log-10 transformed *p*-value of correlation test. The black triangle represents *EGR1*, which shows regulatory effects in both the mouse and the primate datasets. The gray triangles represent the two tested TFs showing regulatory effects in only one dataset (*YY1* and *NFIC*, both from the mouse human-chimpanzee diet dataset). The hollow gray triangles represent the 15 TFs showing no regulatory effect in either dataset (but showing either diet or species effects). (D) Scatter plot of correlation coefficients between *EGR1* and its tested targets, shared by the mouse human-chimpanzee diet dataset and the high-calorie diet dataset. The x- and y-axes show the correlation coefficient between *EGR1* and each target in the high-calorie experiment and the primate diet dataset, respectively. The coefficient of Spearman CoC was 0.34 (*p*-value  = 0.0087). The hollow triangles represent targets showing species effects in the primate dataset.

As mentioned before, TF-mediated differential expression could be associated with dietary differences among species. The human diet is different from the diets of chimpanzees, orangutans, and macaques with respect to multiple fundamental features, including cooking and high calorie and protein contents [3], [13]. If the observed regulatory changes are related to dietary differences, we would expect humans to show the most diverged expression pattern among the four primate species. We indeed found that expression of *EGR1* is significantly elevated in the human liver compared to all other three tested primate species (two-sided t-test *p*<0.05; Figure 2A, left hand panel; Figure S2Bdddd). The expression pattern of *MEF2A* could not be assigned to the human lineage unambiguously: this gene was highly expressed in humans compared to both chimpanzees and macaques, but not with respect to orangutans (Figure S2A). Taken together, these results suggest that up-regulation of *EGR1* liver expression, either hard-wired or plastic, took place on the human evolutionary lineage and led to expression changes of its target genes.

### Transcription factors regulating human vs. chimpanzee diet differences in mouse

If TF-driven regulatory effects observed between human and chimpanzee livers are caused by dietary differences, they might also be detected in mice fed human and chimpanzee diets [19]. To address this, we first determined regulatory effects induced by human and chimpanzee diet differences in mice, and then compared them to those identified in the primate species analysis. The mouse dataset consisted of liver transcriptome data from three groups of six genetically homogeneous male mice, fed a German cafeteria diet, a McDonald's fast-food diet, or a chimpanzee diet (uncooked fruit and vegetables fed to chimpanzees in the Leipzig zoo), for 2 weeks (Figure 1B). Despite considerable differences in their nutritional content, the cafeteria and fast-food diets showed little difference in their effects on liver gene expression [19]. We thus combined the cafeteria and fast-food diet-fed mice in a single ‘human-diet’ group as in the original study. Following the methodology used for the primate dataset, 6,147 genes were detected in this dataset, 1,316 (21.4%) of which were significantly differentially expressed between mice fed human diets and chimpanzee diets (permutation based FDR <10%). At the |effect size|>0.8 cutoff, 2,311 (37.6%) genes showed expression difference between mice fed the two diets.

Comparing the mouse data with the primate data, we found that genes significantly differentially expressed in mice in response to human and chimpanzee diets overlapped significantly with genes differentially expressed between human and chimpanzee livers (one-sided Fisher's exact test, *p* = 0.018). Furthermore, metabolism-related biological processes, including those related to glucose metabolism, showed enrichment among diet-related genes in a Gene Ontology-based analysis (global *p*<0.05, permutation based FDR <10%; Table S2, Table S3, Table S4; Methods). Both results are in accord with the original report [19].

We then identified TFs potentially responsible for gene expression changes induced by human and chimpanzee diets in mice. Among 65 TFs expressed in mice livers, 20 were differently expressed (|effect-size|>0.8) in response to human and chimpanzee diets. These had in total 1,378 potential target genes with an |effect-size|>0.8. Employing the same procedure as applied for the primate dataset, we found that five transcription factors, *EGR1*, *YY1*, *NFIC*, *ATF6* and *RFX1*, showed non-random correlations with their predicted target genes' expression, compared to non-target genes (two-sided Wilcoxon test, *p*<0.01, Table S1). On average, no TF would be expected to show such correlations by chance, as estimated by permuting TF-target relationships (*p*<0.001). Notably, three of the identified TFs have been associated with nutrition and metabolism-associated pathways: *EGR1* expression responds to glucose in blood [28] and to insulin in liver cell lines [29], *YY1* (yin-yang 1) is differentially expressed in the liver between diabetic and non-diabetic rats [30], and *ATF6* (activating transcription factor 6) regulates gluconeogenesis in mice liver [31], supporting the idea that the regulatory effects identified here represent reactions to nutritional change.

### EGR1 is a candidate regulator of diet-related human vs. chimpanzee differences

Remarkably, the single TF showing a human-specific regulatory effect among primates, EGR1, is among the five TFs potentially regulating diet-induced changes in the mouse model. The probability of a TF to randomly show consistent expression differences in primate and mouse datasets is low (one-sided Fisher's exact test, *p* = 0.028; Methods). More importantly, genes up-regulated by EGR1 in humans also tended to be up-regulated in mice fed a human diet, and vice versa (Figure 2A, right hand panel). Specifically, the 23 *EGR1* target genes present in both primate and mouse datasets showed consistent correlations with *EGR1* expression in mouse and primate livers (Spearman *rho*  = 0.46, *p* = 0.028, Figure 2B). Such an extent of TF-target correlation agreement between primate and mouse datasets was not seen for the other two TFs tested in both datasets (*YY1* and *NFIC*; Figure 2Cieiwk.hereieiwk.hereieiwk.hereieiwk.here). Thus, the EGR1-driven differential expression between humans and chimpanzees could be reproduced in mice fed human and chimpanzee diets, suggesting that expression differences between humans and chimpanzees in the liver are partly caused by dietary differences and are regulated through evolutionarily conserved *trans* mechanisms.

One conspicuous difference between human and chimpanzee diets, including those used in the mouse experiment, is the former's high calorie content [13]. To investigate whether the potential EGR1-regulated dietary response may be associated with caloric differences, we analyzed this gene's expression in another dietary manipulation experiment that examined the effects of a high-calorie diet on the mouse liver [32]. This dataset consisted of two groups of 5 individuals fed standard or high-calorie diets for 6 months. We found that *EGR1* was expressed significantly higher in mice fed a high-calorie diet than in those fed the standard diet (one-sided t-test, *p* = 0.007, Figure 2A, right hand panel). Further, *EGR1*-target correlations agreed well with the high-calorie and the human/chimpanzee diet-fed mouse experiments (Spearman *rho*  = 0.34, *p* = 0.009; Figure 2D). Parallel regulatory effects of EGR1 in response to a high-calorie diet and in response to human versus chimpanzee diets in the mouse liver implies a role for EGR1 in coordinating the response to the high caloric content of human diets.

### Computational evaluation of EGR1-target relationships

The 14-bp GC-rich motif recognized by EGR1 (TRANSFAC ID: V$KROX_Q6 [26]; Figure S3) has been derived from 23 EGR1-bound sequences identified in gel shift and DNase I footprinting experiments compiled by TRANSFAC (Table S5). More recently, EGR1 ChIP-chip and ChIP-seq experiments studying blood cell differentiation have supported the authenticity of the EGR1 binding motif described in the TRANSFAC database [33], [34].

Conserved sequences matching the V$KROX_Q6 motif, i.e. the predicted binding sites, occur in total 27 times in the promoters of the 23 predicted targets showing correlated effects between the mouse diet and primate datasets (where the promoter is defined as ±2,000 bp from the TSS). To test the authenticity of these predicted EGR1 binding sites, we conducted four additional analyses. First, we confirmed that these 27 sites are located significantly closer to the TSS rather than being randomly distributed throughout the promoter sequence (one-sided Kolmogorov-Smirnov test *p* = 2.0×10^−5^). The median distance to the TSS was found to be 392 bp (Figure S4). Second, we asked whether the motifs might randomly occur due to the promoters' dinucleotide content and overall conservation. To address this, we applied the binding site prediction algorithm on randomly shuffled sequences of the promoter regions of the 23 predicted targets, while keeping the average conservation of each nucleotide type and the dinucleotide content fixed for each target gene (Methods). The results showed that on average only one gene would pass the original criteria for being predicted as EGR1 target by chance (FDR  = 4%). Third, we investigated whether the predicted binding sites overlap with DNase I-hypersensitive sites, regions of open chromatin generally bound by TFs. Using published data from 15 cell lines including human liver carcinoma cells [35] (Methods), we found that 22 of the 23 common targets contain at least one predicted EGR1 binding site overlapping a DNase I-hypersensitive site. This is highly unexpected, as gauged by randomly choosing binding sites with the same length and comparable G/C content as the predicted binding sites in the 23 promoters (permutation test, *p*<0.001). Fourth, we tested whether the predicted EGR1-target relationships can be reproduced using an independent liver gene expression dataset comprising a large sample of healthy humans [36]. We found significantly better correlation between the expression of *EGR1* and its targets identified in our study, than between *EGR1* and non-target genes, as well as between *EGR1* and its other predicted targets (Wilcoxon test *p*<0.002; Figure S5; Methods). Taken together, these results suggest that the majority, if not all, of these 23 genes are regulated by EGR1.

### Mouse-specific responses to human vs. chimpanzee diet differences

**Figure 3:**
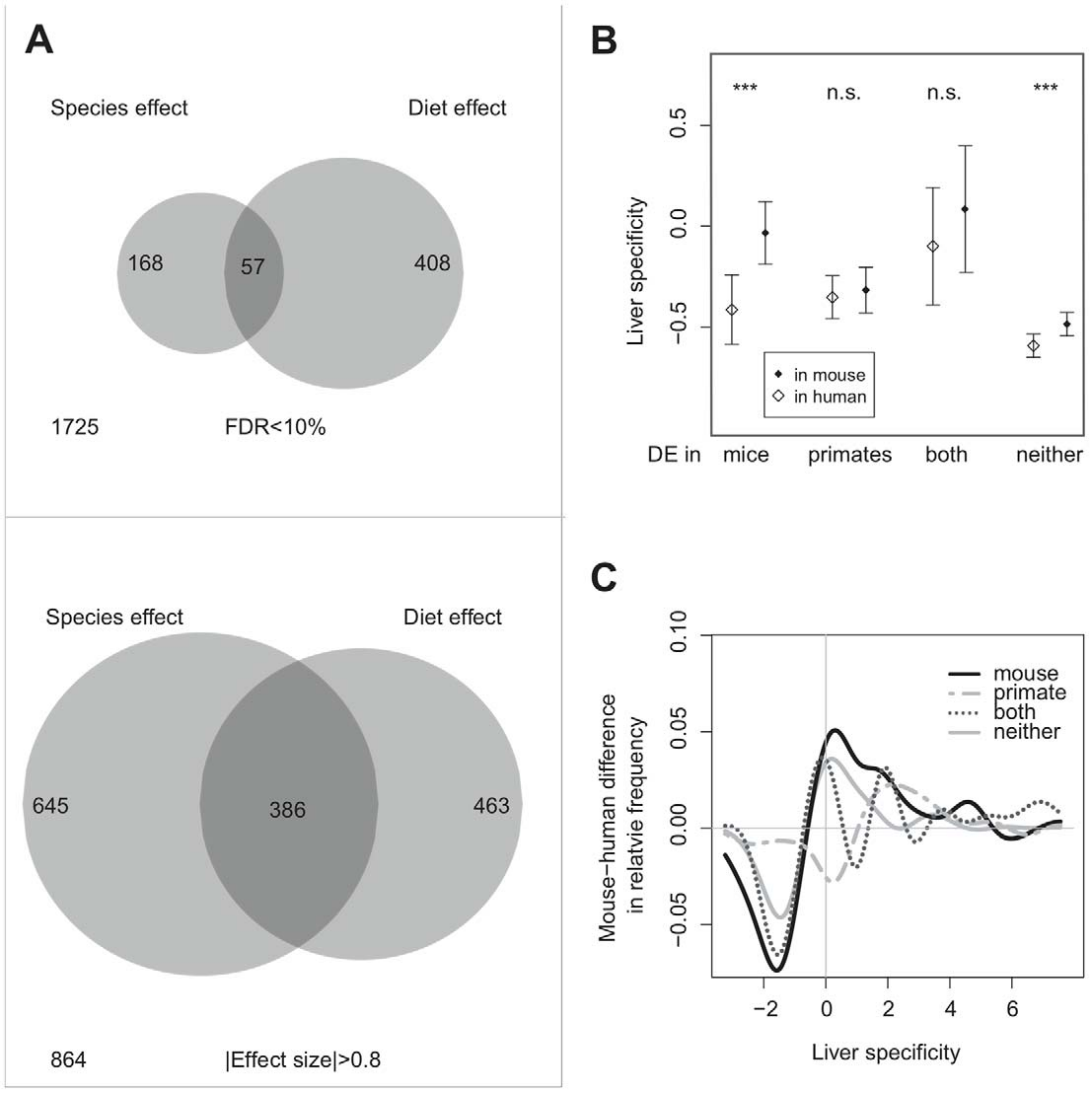
Mouse-specific responses to human-chimpanzee dietary differences. (A) Venn diagram showing the numbers of human-mouse orthologous genes differentially expressed between mice fed human and chimpanzee diets in liver (left), and genes differentially expressed between human and chimpanzee livers (right). Top panel: genes showing differential expression at a stringent cutoff, FDR <10% in each of the two primate datasets and the primate diet dataset; lower panel: genes showing diet/species effects at a loose cutoff, |effect size|>0.8. Numbers outside the circles indicate orthologous genes showing no species or diet effects. Only genes detected in both primate and mice datasets are represented. Note that upon relaxing the differential expression cutoff, the number of genes showing species effects increases by ∼5 times, while those showing diet effects increases by ∼2. This is caused by differences in the distribution of effect size and statistical power between the two datasets (Figure S5). In the mouse dataset, small effects are more easily detected as statistically significant, likely due to lower within-group variance. (B) Median transcriptional liver-specificity among different groups of genes. Liver-specificity is calculated as the difference between liver expression and mean gene expression level across various tissues, in units of standard deviation (i.e. a z-score). Shown are four groups of genes that were differentially expressed only between mice fed human and chimpanzee diets, only between human and chimpanzee, in both primates and mice, or in neither. Black diamonds show median liver-specificity in mouse; white diamonds show liver-specificity in human (using data specific to each species). The range of whiskers is M±1.58×IQR/n^0.5^, where M, IQR and n are the median, interquantile range, and number of observations. Asterisks indicate significance based on two-sided Wilcoxon test. ***: *p*<0.001. n.s.: *p*>0.1. (C) The difference between mouse- and human liver-specificity distributions, across the same gene sets as in panel B. The mouse and human distributions were each converted into Gaussian kernel densities (estimated using the “density” function in R); the y-axis shows the difference between these densities. The x-axis shows liver-specificity as in panel B. For example, positive x- and y-axis values indicate that the mouse shows an excess of genes showing high liver-specificity, compared to human. Black solid line: Genes differentially expressed only in mouse; double-dashed gray line: only between human and chimpanzee; gray dotted line: in both mouse and primates; gray solid line: in neither. While genes differentially expressed in neither dataset have higher mouse liver-specificities relative to human, this is significantly more pronounced among mouse-specific differentially expressed genes (one-sided Wilcoxon test, *p* = 0.0077; Methods), and is not seen for the primate-specific differentially expressed genes.

In addition to parallel expression differences between mice and primates, our analysis revealed substantial differences in expression response to diet between the two taxa. Specifically, among 2,358 orthologs expressed in both datasets, 57 genes were differentially expressed in both mice and primates at the FDR <10% cutoff (requiring FDR <10% in each primate dataset; Methods) (Figure 3A). In contrast, 168 genes were differentially expressed only between humans and chimpanzees, and 408 genes only between mice fed human and chimpanzee diets. Differential expression observed between humans and chimpanzees, but not between mice fed human and chimpanzee diets, may have various explanations. These could include other environmental differences between these species, as well as neutral gene expression divergence caused by accumulation of *cis* differences [37]. In contrast, the majority of expression differences found in genetically homogeneous mice living in a controlled environment should represent mouse-specific responses to a single environmental variable: diet. Studying these differences should help understand how species diverge in their responses to the same environmental change.

The 408 genes showing mouse-specific dietary response constitute 17% of all orthologs expressed in both the mouse and the primate livers. With respect to function, these genes were significantly enriched in carbohydrate metabolism-related functions (*p*<0.05, FDR <10%, Table S2, Table S6). This is important, as it suggests that the mouse-specific responses are indeed due to dietary effects.

What could cause these dietary responses to be observed in mice fed human and chimpanzee diets, but not between humans and chimpanzees? One possibility is that the orthologous primate genes do respond to diet, but at weaker levels, and that we lacked the statistical power to detect differential expression, due to technical or biological reasons, when testing each gene individually. If so, we might expect human-chimpanzee expression divergence across these 408 genes, as a group, to be greater than expression divergence across other expressed genes. However, we found no such tendency (Figure S6). This indicates that the mouse-specific diet effect was not caused by a lack of power in the primate dataset.

May differences between mouse and primate dietary response be caused by the transient nature of dietary exposure in the mouse experiment [19]? Arguing against this, we detect the same *EGR1-*regulated expression response in mice fed human diets for 2 weeks, and in mice fed high-calorie diets for 6 months. Likewise, it has been shown that a two week period is sufficient for mice fed a high-fat diet to reach stable plasma levels of total and LDL cholesterol [38].

Finally, it is possible that these genes acquired novel functions in the primate or the mouse liver through changes in their protein structure. However, we found no indication of faster amino acid sequence evolution among these 408 genes compared to other genes expressed in both the primate and the mouse datasets (using dN/dS ratios between humans and chimpanzees or between humans and mice; one-sided Wilcoxon test *p*>0.1). Instead, we found that primates and mice differ in how these genes are regulated across tissues. Specifically, transcriptional liver-specificity (gene expression level in liver relative to its expression across 79 or 61 tissue types in human and mice, respectively [39]) among these 408 genes was significantly higher in mice than in humans (one-sided Wilcoxon test, *p* = 0.008, Figure 3B–C). This was not seen among, for instance, genes differentially expressed in *both* mice and primates. In other words, the expression of the 408 genes has become more liver-specific in mice, or less liver-specific in humans (and potentially also in other primates). The reason for this change is not related to an overall change in the average expression level of the 408 genes in humans: the 408 genes were expressed at similar levels between the human liver and the mouse liver (Figure S7). These observations were stable at an alternative effect size cutoff, indicating the robustness of the result (Figure S8, Figure S9 and Table S7).

Mechanistically, how could these genes show more liver-specific expression in mouse and respond to dietary change only in this lineage? Notably, four of the five TFs identified as potential regulators of the response to human-chimpanzee diet differences in the mouse liver, were either not differentially expressed between human and chimpanzee livers (*YY1* and *ATF6*) or oppositely differentially expressed between primates and mice (*RFX1* and *SREBF1*; i.e. humans and human diet-fed mice showed contradictory effects) (Table S1). In addition to these *trans-* changes, we also found that the proximal promoter and the 3′ un-translated region (3′ UTR) sequences of the 408 genes were significantly less conserved among placental mammals compared to the other expressed genes (one-sided Wilcoxon test, *p*<0.002; Methods). This suggests that both *trans-* and *cis-*regulatory changes controlling the expression of these genes may have led to differential tissue specificity between mice and primates, and eventually, differences in diet-related response between the two lineages.

## Discussion

Our results show that a subset of gene expression differences between human and chimpanzee livers may be regulated through differences in expression of a single TF: *EGR1*. Using orangutan and rhesus macaque expression as outgroup references, we predict that an increase in the expression level of *EGR1*, as well as its predicted target genes, took place on the human evolutionary lineage after the human-chimpanzee lineage split (see below). We further demonstrate that elevated expression of *EGR1* and its predicted targets observed in the human liver is replicated in mice fed human versus chimpanzee diets, and in mice fed high calorie versus low calorie diets. This implies that the human-specific increase in *EGR1* expression represents a response to the high caloric content of the human diet.

### EGR1 function and target recognition

The immediate early gene EGR1 is a versatile zinc-finger type transcription factor, functioning in diverse tissues (reviewed in [40]). For example, it plays inhibitory/excitatory roles in the growth of different cancer types [41], [42], while in the brain it has a role in memory formation [43]. Notably, EGR1 was previously shown to play a role in dietary lipid response in mouse [44], in insulin response in rat liver cells [29], and in glucose response in human mononuclear cells [28], consistent with a role for EGR1 in a potentially adaptive response to the human-specific high-nutrient diet.

There is a total of 11,123 occurrences of V$KROX_Q6 motifs in promoter regions across the genome, and 1,267 (∼12%) of these show conservation across vertebrates (Methods). Among the 1,267 genes containing such conserved binding sites, 333 and 372 were expressed and 45.6% and 15.3% of these showed expression correlation with *EGR1* in the primate and mouse diet experiments, respectively (Pearson correlation test *p*<0.05). Recent ChIP-chip and ChIP-seq experiments studying EGR1 activity during blood cell differentiation have likewise identified thousands of binding sites across the human genome [33], [34], suggesting a wide spectrum of targets regulated by EGR1.

Importantly, EGR1 has been reported to recognize two distinct motifs [45], described in the TRANSFAC database as V$EGR1_01 and V$KROX_Q6. We found significant non-random correlations between *EGR1* and targets predicted based on V$KROX_Q6 only. This may not be unexpected, given that the two motifs regulate distinct sets of genes during blood cell differentiation [46]. Meanwhile EGR3, which recognizes the same motif and is differentially expressed in the primate dataset, does not show significant non-random correlation with its predicted targets' expression (Wilcoxon test *p*>0.1).

### Differential regulation of *EGR1* between human and chimpanzee

In mice, elevated *EGR1* expression was caused by an environmental change in dietary content. What mechanism could be driving *EGR1*'s differential expression between human and chimpanzee livers? Two scenarios are possible. First, the *EGR1* effect in the human liver might be purely environmental, that is, a plastic response to differences in caloric content of the study subjects' diet, as seen in mice. Second, continuous exposure to a high-calorie diet during human evolution might have led to genetic assimilation [25], that is, selection and fixation of genetic changes that permanently elevated *EGR1* levels in the human liver. Under the latter scenario, mutations causing elevated *EGR1* expression in the human liver may, in addition, result in similar expression increases in other human tissues. Indeed, gene expression differences linked to *cis*-regulatory mutations can readily penetrate multiple tissues (e.g. [47]), whereas plastic responses to environmental changes might be expected to involve specific tissues. For instance, the majority of expression changes observed in the livers of mice fed human versus chimpanzee diets, were not observed in the mouse brain [19].

To test a possible *cis*-regulatory effect permeating multiple tissues, we analyzed *EGR1* expression in published human and chimpanzee gene expression data including brain, testis, kidney, and heart [20]. We found that *EGR1* showed higher average expression in kidney and testis, including significantly higher expression in the latter (*p*<0.05, Figure S10). This result raises the possibility that *EGR1*'s differential regulation in the human liver is not restricted to this tissue and, therefore, might be caused by *cis*-regulatory mutations. The differential expression pattern found in brain and heart could then involve *trans-*acting tissue-specific regulation or the use of alternative promoter regions [48]. That said, the evidence is equivocal, and further work is needed to resolve the mechanism of *EGR1* differential expression between human and chimpanzee livers.

Meanwhile, the expression pattern of EGR1 targets among primate species differs from primate EGR1 expression and from EGR1 targets' expression in the mouse diet experiment (Figure 2A). The primary inconsistency involves the high expression of EGR1 targets in orangutans. Considering that orangutan diets in captivity are more similar to that of chimpanzees than humans, this result raises doubt on whether the predicted targets are all responsive to dietary differences. We note, however, that our statistical power to accurately measure expression in orangutans is particularly limited. Indeed, the combined dataset comprises 18 human and 17 chimpanzee samples, compared to 12 macaque and only five orangutan samples. In addition, orangutan expression profiles were measured only by human microarrays and might have low reliability given the evolutionary divergence between humans and orangutans. The expression levels in macaque samples were measured by the RNA-sequencing, and human and chimpanzee samples on both platforms; data for the latter species should therefore be more reliable.

### Additional regulators of dietary response

EGR1 is unlikely to be the only regulator involved in dietary adaptations common to all humans. First of all, current knowledge of TFs and TF-based transcriptional regulation is greatly limited by incomplete annotation. More than a thousand human genes are assigned transcription factor activity in the Gene Ontology database [49], but less than 300 have annotated target recognition sites [26]. Furthermore, many TF binding sites are short and degenerate, leading to high false discovery rates in TFBS prediction [50]–[52], and constraining our power to estimate TF regulatory effects.

In addition, here we have not considered other important classes of gene expression regulators, such as microRNA [53], histone modifiers [54], or DNA methyltransferases [55]. As transcriptional regulation is a combined effect of multiple factors, measuring and incorporating the effects of these regulators should lead to substantially improved models of differential expression between species.

It is also worth noting that we could associate only 39% of genes (n = 814) responding to human and chimpanzee dietary differences in mice with the expression variation of their putative TF regulators. In other words, mechanisms driving the majority of diet-related expression changes in mice also remain unknown. Further studies are needed to identify transcriptional regulators involved in conserved and human-specific dietary responses across mammalian species.

### The utility of mouse models

Mice have been used to study diverse biological phenomena relevant to humans, including diet-related diseases such as type 2 diabetes [17] and obesity [38]. The relevance of mouse models for studying differences between human and other primates' diets, however, is not well understood. Here we found evidence for extensive mouse specificity in response to primate dietary differences: as much as 88% of strong gene expression changes observed in mice, in response to human and chimpanzee diets, were not observed in primates. Our results further imply that this high degree of mouse-specificity in the dietary response could be due to altered transcriptional tissue specificity of the genes involved. These findings raise caution with regard to the interpretation of results from mouse dietary response models and their extrapolation to humans.

Future work with mice subjected to longer-term exposure to particular nutritional content could reveal the exact limitations of mouse models for studying the molecular basis of human dietary change. Meanwhile, alternative models with closer genomic similarity to humans, such as pigs or primates, may prove more effective than mice for this purpose.

## Methods

### Microarray and RNA-sequencing datasets

We used two previously published gene expression datasets of postmortem primate liver. One was based on Affymetrix Human Genome U133plus2 GeneChip® arrays measured in six humans, five chimpanzees and in five orangutans [20], [21], available at the ArrayExpress Archive (http://www.ebi.ac.uk/arrayexpress/) with accession numbers E-AFMX-11 and E-TABM-84. A second dataset was based on RNA-sequencing on the Illumina platform, which contained data from 12 humans, 12 chimpanzees and 12 rhesus macaques [22] and was downloaded from NCBI Gene Expression Omnibus (GEO) public data repository [56] (http://www.ncbi.nlm.nih.gov/projects/geo/query/acc.cgi?acc=GSE17274).

We also used a mouse liver gene expression dataset, where expression was measured using Mouse Genome 430 2.0 GeneChip® arrays in 24 mice fed two human diets, one chimpanzee diet, and one regular mouse food diet, six mice for each diet [19], downloaded from GEO (http://www.ncbi.nlm.nih.gov/geo/query/acc.cgi?acc=GSE6297).

### Preprocessing gene expression data

For Affymetrix microarray data analysis, we summarized expression levels per Ensembl [57] gene (version 54) using custom CDF files [58] (available at http://brainarray.mbni.med.umich.edu/Brainarray/Database/CustomCDF/genomic_curated_CDF.asp). Expression levels were calculated using the “rma” (robust multichip average) method in the “affy” package [59], which is part of the R Bioconductor software [60]. Microarray probes that did not match human (hg18), chimpanzee (pantro2) and orangutan (ponabe2) genomes perfectly were identified using BLAT [61] and discarded. The extracted expression levels were log transformed and quantile-normalized. Detection *p*-values (probability of the expression signal representing background) were calculated using the “mas5” method in the same package. In further analysis we included (1) genes with a nominal detection *p*-value <0.05 among at least half of the samples, and (2) genes showing above-detection expression unevenly among sample groups (indicating differential expression), as determined using the “dMFNCHypergeo” method in the R “BiasedUrn” package [62] at *p*<0.1.

For the RNA-sequencing dataset, preprocessed read counts for 20,689 Ensembl genes were directly downloaded from NCBI GEO with accession GSE17274. 7,544 genes that had no read count in more than half of all samples, or which had no differential expression test *p*-value according to the “DESeq” R package (see below) due to 0 variance, were removed from further analyses, resulting in 13,145 genes. Read counts were log transformed and quantile-normalized.

### Choice of statistical tests

We used parametric tests for testing differential expression (t-test or ANOVA) or comparing expression profiles between pairs of genes (Pearson correlation test). When comparing distributions of variables that are by definition not normally distributed (e.g. correlation coefficients or dN/dS ratios) we used non-parametric tests (Spearman correlation and Wilcoxon signed-rank tests).

### Testing for differential gene expression in microarray datasets

We used ANOVA to test for each gene's differential expression among groups. If data were generated in different batches, two-way ANOVA was used with experimental batch (the day of hybridization) included as an additional factor. To remove the batch effect, for each gene, we subtracted each batch's mean from expression profiles of samples within that batch. If a factor (e.g. species) had more than two levels, testing differential expression between each pair of levels was accomplished using the Tukey HSD *post hoc* test (“TukeyHSD” function from the “stats” R package [60]).

### Testing for transcriptome-wide human vs. chimpanzee differences

We first determined differential expression in each primate dataset separately, and then combined the results. To identify differential expression in the RNA-sequencing dataset, we first determined a *p*-value cut-off based on a permutation approach to ensure FDR <10%. Specifically, (1) species identities of samples were randomized, (2) a differential expression test (using the “nbinomTest” method in the “DESeq” R package [63]) was applied to all genes using the randomized species identities. This routine was repeated for 1,000 times, and the exact p-value cutoff was chosen to ensure that the median number of significant genes across the 1,000 permutations is 10% of the actual number of significant genes. Using this criterion, we identified 4,551 out of the 13,145 genes as differentially expressed between human and chimpanzee at *p*<0.024.

For the Affymetrix U133plus2 dataset, using a similar procedure we identified t-test *p*<0.037 as the cutoff at FDR <10%. At this cutoff, 969 genes were differentially expressed between humans and chimpanzees out of 4,531 expressed genes.

The two datasets were combined using 4,161 genes that were commonly expressed in both, with each gene's expression profile standardized (mean extracted and then divided by the standard deviation, resulting in a z-score) first across human and chimpanzee samples in each dataset and subsequently across all samples from both datasets. 428 genes that showed significant differential expression (at FDR <10%) in both datasets in the same direction (e.g. higher in human in both datasets) were considered as significantly differentially expressed between human and chimpanzee. In addition, we defined an effect size-based set of genes showing species effects (see below).

### Testing for transcriptome-wide diet effects in mouse

Following the original mouse diet study, we combined the data from the two human diets – the cafeteria and fast food diets – due to their similar effects on mouse liver gene expression levels, and compared them directly to the chimpanzee diet. Using the permutation-based approach described above, 1,316 out of 6,147 expressed genes showed differential expression between mice fed human and chimpanzee diets at ANOVA *p*<0.073 with FDR<10%.

### Effect size calculation

The following formula was adopted to calculate effect size: *d* = (M_1_–M_2_)/SD_p_, where M_1_ and M_2_ are the means of the two groups and SD_p_ is the pooled standard deviation, calculated as (((N_1_−1)×SD_1_^2^+((N_2_−1)×SD_2_^2^))/(N_1_+N_2_−2))^0.5^, where N_1_ and N_2_ are the sample sizes and SD_1_ and SD_2_ are the standard deviations of the two groups, respectively. In the mouse and primate datasets, the first group was mice fed human diets or humans, the second mice fed chimpanzee diets or chimpanzees, respectively.

### Rational for using effect size as cutoff

In addition to ANOVA, we also used effect size to define differentially expressed gene sets, for two reasons: First, using effect size allows straightforward comparisons of datasets with different sample sizes. Second, the number of genes reaching nominal significance cutoffs for differential expression in both the primate and mouse datasets was limited (N = 57), too small to allow testing for common regulatory factors. We reasoned that this narrow overlap might partly be caused by weak statistical power to detect differential expression in either dataset (i.e. high false negative rates). One approach to overcome this limitation is to search for regulatory effects over a larger set of genes showing weaker differential expression signals. We thus chose a more relaxed cutoff to determine species or diet effects based on effect size (|effect size|>0.8). The cutoff 0.8 has been proposed as a general cutoff for modest effects [64]. Note that in the combined primate dataset, this cutoff roughly corresponds to t-test *p*-value <0.025 (permutation based FDR  = 4.4%).

### Gene Ontology analysis

We used the Gene Ontology (GO) [49] and the Fisher's exact test for functional analysis. Annotations from the biological process (BP) ontological domain were used. Ensembl genes with GO annotation downloaded from Ensembl (version 64) were assigned to GO categories based on Ensembl GO annotation and the Gene Ontology directed acyclic graph (DAG), accessed through the “GO. db” R package [65] (this latter step is necessary to assign genes to ancestral GO categories, which are not included in the Ensembl table). The numbers of tested genes and those of their relevant background genes with annotations are shown in Table S2. Genes expressed in a dataset but that did not show a specific effect, were chosen as background. Only GO categories containing a minimal number of genes with GO annotation were tested (see Table S2). To correct for multiple testing, we randomly re-sampled the same number of genes as in the tested set from the relevant background genes with GO annotation for 1,000 times. The FDR was defined as the ratio of the expected (median) relative frequency of significantly enriched categories among the 1,000 permutations, to that observed, at a certain *p*-value cutoff. The global significance of the tests across all GO categories was defined as the relative frequency of permutations with at least as many enriched categories as that observed, passing a *p*-value cutoff. When reporting significance, we use a *p*-value cutoff (chosen from 0.05, 0.01, 0.005, and 0.001) at which FDR <10% and the global *p*-value <0.05.

### Predicting target genes of transcription factors

We borrowed the procedure from [27] to predict target genes of each transcription factor (TF). Briefly, the “MATCH” algorithm from the TRANSFAC database (version 7.1) [26] was used to predict TF binding sites (TFBS) on each gene's putative promoter region; genes with at least one conserved predicted binding site of one TF were considered that TF's targets. Specifically, the promoter was defined as the region within 2,000 base pairs both upstream and downstream of the focal gene's TSS (as annotated by Ensembl version 54 [57]). To find TFBS conserved among vertebrates, we required that ≥80% of nucleotides of the focal TFBS have 17-way vertebrate PhastCons scores and an average score ≥0.6. PhastCons scores were obtained from the UCSC Genome Browser 17-way Vertebrate Conserved Element Table [66].

### Identifying candidate transcription factors regulating expression differences

We used the same procedure for identifying candidate TFs regulating observed differential gene expression between groups, in both the primate and mouse diet datasets. Briefly, we first narrowed the search space to TFs and predicted target genes showing differential expression, and then tested each TF for non-random (more positive or more negative) correlations with its targets, compared to non-target genes (genes that are targets of other TFs). This was considered indication of a regulatory effect of the TF on its targets. Specifically, we calculated Pearson correlations between each TF and its predicted target genes that showed at least minimal species or diet effects (|effect size|>0.8). These correlations were then compared to that between the same TF and non-target genes whose |effect size|>0.8, using a two-sided Wilcoxon test (given that correlation coefficients are not normally distributed). A *p*-value <0.01 was used as cutoff. When a TF was associated with more than one TFBS motif (8 cases in the primate dataset and 9 in the mouse primate diet dataset), we tested target gene sets for each motif separately. To estimate how many TFs would pass the cutoff randomly, TF-target relations were permuted 1,000 times, the above-procedure applied each time, and the number of TFs passing the cutoff recorded. The global significance was defined as the relative frequency of permutations with the same number or more TFs passing the cutoff as that observed.

### Testing for excess of common candidate TFs in the mouse and the primate datasets

When choosing TFs that were detected in both the combined primate dataset and the mouse dataset (N = 36) as background, we performed a one-sided Fisher's exact test for the overlap between candidate TFs from each dataset that showed consistent changes in the primate and mouse diet datasets: e.g. up-regulation in humans or under a human diet in mice (note that *EGR1* was the only TF showing consistent change).

### Testing for correlation of TF-target correlations between mice and primate datasets

The predicted TF-target relationships were permuted for genes that showed at least minimal species or diet effects (|effect size|>0.8) in both the mouse and primate datasets. For each TF that was identified as candidate regulator in at least one dataset, we calculated its correlation with its tested target genes in both datasets. Next, the correlation of these correlations (CoC) between two datasets was calculated using Spearman correlation (given that correlation coefficients are not normally distributed). This procedure was applied 1,000 times, and the relative frequency of random cases in which the number of TFs whose CoCs were no less significant than observed (i.e. for *EGR1*) was used as measure of significance. The procedure was repeated also for *YY1* and *NFIC*, the two TFs that showed a diet effect and regulatory effects and diet in the mouse dataset, and species differences in the primate dataset.

### The high-calorie mouse diet dataset

A mouse liver gene expression dataset based on Agilent-012694 Whole Mouse Genome G4122A (Feature Number version) containing liver samples from mice fed normal or high-calorie diets (5 individuals per group) was downloaded from NCBI GEO with accession number GSE6089. The data analyses followed the same procedures described above.

### Non-random occurrence of conserved EGR1 binding sites

To test whether the occurrence of conserved EGR1 response motifs (V$KROX_Q6) in the 23 common targets' (genes identified as targets in both the mouse diet and primate datasets) promoter regions may reflect the nucleotide composition of these promoters, we performed a randomization test while controlling for overall G/C or dinucleotide content and conservation. Specifically, using the uShuffle software [67], we randomized the proximal promoter sequences (±2,000 bp from the TSS) while keeping the numbers of all possible 16 dinucleotides fixed. We further permuted PhastCons scores per nucleotide, while keeping the distribution of PhastCons scores among the 4 nucleotide types fixed. We thus generated one thousand batches of random sequence, for each of the 23 genes' promoter regions. The sequences were used as input in the TRANSFAC “MATCH” algorithm [26]. In each batch, we asked whether each of the 23 genes would be predicted as EGR1 target, i.e. whether it contained at least one binding site fulfilling the same criteria as in the original analysis. The maximum number of genes containing at least one predicted EGR1 binding site among 1,000 random batches was 4 (Figure S11). The random expectation was calculated as the median of this distribution.

### Overlap between binding sites and DNase I hypersensitive sites

Processed DNase I footprint data from human lymphoblast cell lines as well as 14 cell lines generated by the ENCODE project [68] were obtained from http://centipede.uchicago.edu/SimpleMulti/ [35] and used conforming to the ENCODE Consortium Data Release Policy [69]. DNase I sites cover ∼6% of the 23 common EGR1 targets' promoters (genes identified as targets in both the mouse diet and primate datasets). In 22 of the 23 promoters, we found a minimum 1-nt overlap between a DNase I site and the conserved EGR1 binding site. To calculate the random expectation we used the following procedure: (1) For each of the 23 genes, a 14-nt long DNA stretch (the length of the EGR1 response motif) was randomly chosen from the gene's proximal promoter sequence and its G/C content was calculated. (2) This procedure was repeated until 1,000 14-nt sequences of comparable G/C content were chosen. We required that the G/C content of the sequences to be at least as high as that of the originally identified 27 binding sites (79%). We thereby controlled the occurrence of high G/C content within DNase I hypersensitive sites. (3) The number of random sequences with a minimum of 1-nt overlap with DNase I sites was calculated. The *p*-value was defined as the relative frequency among the 1,000 randomizations in which the number of genes with a binding site-DNase I site overlap was equal to or larger than that observed (i.e. 22 genes). The random expectation (median number of genes with overlap among the 1,000 randomizations) was calculated as 11/23.

### Correlation between *EGR1* and the 23 common targets in human liver

Liver transcriptome data from a large human sample (N = 60, each with two replicates) was downloaded from NCBI GEO with accession number GSE28893 [36]. The data was quantile-normalized. In this dataset 13,942 genes were expressed, including 20 of the 23 common targets. A one-sided Wilcoxon test was used to test if these 20 genes were more strongly correlated with EGR1 than (i) other expressed genes predicted as EGR1 target based on sequence predictions, (ii) all other expressed genes with TF annotation.

### Human vs. chimpanzee divergence rate at putative promoter regions and protein coding DNA sequence

We used human-chimpanzee promoter divergence rates (Kp), normalized by an estimate of the substitution rate of a genomic region (Ki), as calculated by [20]. This measure was used to estimate sequence divergence for promoter regions for human and chimpanzee. Divergence at protein coding regions was defined by non-synonymous divergence normalized by synonymous divergence (dN/dS), and was downloaded from the Ensembl database (version 60).

### Mammalian conservation at promoter and 3′ UTRs

We used PhastCons scores to estimate sequence conservation as previously reported [27]. Briefly, using the PhastCons 18-way Placental Mammal Conservation Track (a subset of the 28-way Placental Track) from the UCSC Genome Browser, for each Ensembl human gene, we calculated mean sequence conservation for proximal promoter (±2,000 bp from the TSS) and 3′ UTR.

### Calculating liver-specificity in gene expression

A dataset including 79 human tissues and 61 mouse tissues [39] was used to calculate each gene's expression level in liver relative to that in other tissues. Specifically, for each species, for each gene, the liver expression level was scaled as the distance to the mean in units of standard deviation across all tissues, i.e. a z-score. We compared liver-specificity among gene sets using this z-score.

### Testing liver-specificity differences among gene sets

To estimate the significance of the difference between human and mouse in liver-specificity among genes differentially expressed only in the mouse dataset (the foreground genes), we first had to account for overall differences in liver-specificity between the two species, which could arise because of technical or sampling reasons. To achieve this, we normalized the liver-specificity measure using a background gene set that should not show difference in liver-specificity: genes that were differentially expressed neither in mice nor in primates. We shifted the human and mouse measures so that the background genes had the same median liver-specificity in both species. We then tested for a higher median of these foreground genes' liver-specificities in mouse than in human using a one-sided Wilcoxon test (*p* = 0.0077).

## Supporting Information

Figure S1 **Consistency between microarray and RNA-sequencing datasets of primate liver gene expression.** Scatter plot of effect sizes of differentially expressed genes (permutation based FDR <10%). Gray: genes differentially expressed in either primate dataset. Black: genes differentially expressed in both primate datasets. Red: genes differentially expressed in both primate datasets and in the mouse diet dataset.
(TIFF)

Figure S2 **Liver expression profiles of** ***MEF2A*** **and** ***EGR1*** **among primates.** The two TFs, *MEF2A* (A) and *EGR1* (B), were chosen for showing significantly non-random correlations with their predicted targets, where both the TFs and targets also showed species effects (|effect size|>0.8). Shown are relative expression levels (distances to mean levels in units of standard deviation across all liver samples) of the two genes in human, chimpanzee, orangutan and macaque samples, based on the microarray (‘Array’) and RNA-seq datasets. Results are expressed as mean ± SEM. *EGR1* expression was significantly higher in humans compared to all other primates based on both the RNA-seq and the microarrays dataset (one-sided t-test *p*<0.05).
(TIFF)

Figure S3 **Sequence logo of V$KROX_Q6.** This is the logo of V$KROX_Q6 created using WebLogo [70]. The sequence from the 6^th^ to 10^th^ position (GGGGG) is defined as the core element by TRANSFAC. Note that TRANSFAC arbitrarily chose sequences from the strand opposite to the one bound by EGR1 and uses a motif reverse complementary to this one (personal communication).
(TIFF)

Figure S4 **Positions of 27 binding sites of the 23 common genes.** The x-axis is the distance to transcription start site (TSS). Positive values are downstream (3′ direction) to TSS and negative upstream.
(TIFF)

Figure S5 ***EGR1-*** **target gene correlations in the human liver.** The boxplots represent absolute value Pearson correlation coefficient distributions between EGR1 and three gene sets in a human liver dataset [36]. The gene sets are (a) the common EGR1 targets identified in the primate and mouse diet datasets (20 of the 23 genes expressed in the human liver dataset); (b) all predicted targets based on evolutionary conservation of the TFBS (n = 495); (c) all other expressed genes annotated as TF targets (n = 7,348). Asterisks indicate significance based on one-sided Wilcoxon tests, **: p<0.01. Note that we use absolute correlation coefficients because a number of observations suggest that EGR1 may act both as an activator and repressor of transcription.
(TIFF)

Figure S6 **Mouse-specificity of dietary response is not due to lack of power.** Boxplot of human vs. chimpanzee liver expression |effect sizes|, among genes showing or not showing diet effects in mice. “DE in mouse” indicates the 408 genes that were significantly differentially expressed (FDR <10%) only between mice fed human and chimpanzee diets but not between humans and chimpanzees in liver, “DE in neither”indicates the 1,725 genes that were differentially expressed neither between mice fed human and chimpanzee diets nor between humans and chimpanzees in liver. n.s: two-sided Wilcoxon test *p*-value>0.1.
(TIFF)

Figure S7 **Mouse-specific genes show higher liver specificity but not higher expression levels in mouse liver.** Boxplot of relative expression levels in mouse and in human livers (distance to the mean level in units of standard deviation across all expressed genes within liver), across mouse-specific differentially expressed genes (two-sided Wilcoxon *p*>0.1).
(TIFF)

Figure S8 **Distributions of absolute effect sizes of commonly detected genes.** Shown are distributions of absolute effect sizes (|effect sizes|) of the 2,358 genes that were detected in both the primate dataset and the diet dataset. The gray and pink bars show the effect size at the FDR <10% cutoff in the mouse and at the cumulative FDR <5% in the combined primate datasets, respectively. The effect size cutoff 0.8, used in identifying regulatory effects in both datasets, is shown in green.
(TIFF)

Figure S9 **Mouse-specific dietary response under different cutoffs.** To ensure that our results on mouse-specific dietary response were not affected by the choice of statistical cutoff, we repeated the analyses using an effect size cutoff (|effect size|>1.13) in the primate dataset equivalent to FDR <10% in the mouse diet dataset. (A) Boxplot of human vs. chimpanzee liver expression |effect size|, among genes showing or not showing diet effects in mice. “DE in mouse” indicates the 321 genes that were differentially expressed only between mice fed human and chimpanzee diets but not between human and chimpanzee in liver, “DE in neither” indicates the 1,392 genes that were differentially expressed neither between mice fed human and chimpanzee diets nor between human and chimpanzee in liver (see Figure S3). Here and in panel B–C, n.s: two-sided Wilcoxon test *p*>0.1; ***: *p*<0.001. (B) Boxplot of relative expression levels (distances to mean levels in units of standard deviation across all expressed genes within liver) of the 321 mouse-specific differentially expressed genes in human and mouse livers (two-sided Wilcoxon *p* = 0.47, see Figure S4B). (C–D) Liver-specificities of the 321 genes in human and mouse. Significance in all tests remains qualitatively similar to results shown in Figure 3. The difference in liver-specificities of mouse-specific differentially expressed genes between human and mouse after correcting human-mouse liver specificities of background genes is still significant (one-sided Wilcoxon test, *p* = 0.007). Compare to Figure 3.
(TIFF)

Figure S10 **Expression divergence of** ***EGR1*** **between human and chimpanzee in five tissues.** Median expression of *EGR1* in five tissues (brain, heart, kidney, liver and testis) [20]. The boxplots were plotted using the “boxplot” function in the R ‘graphics’ package [42]. Under default settings, the whisker ranges are calculated as: M±1.58×IQR/n^0.5^, where M, IQR and n are the median, interquantile range, and number of observations. Human-chimpanzee effect sizes are shown at the bottom line.
(TIFF)

Figure S11 **Distribution of numbers of predicted EGR1 targets based on random promoter sequences.** Number of genes predicted as EGR1 targets based on random promoter region sequences shuffled maintaining dinucleotide levels (see Methods). The same criteria were used for the real promoter region sequences. The median of the distribution was found to be one.
(TIFF)

Table S1 **Candidate TFs driving differential gene expressions between human and chimpanzee, or between mice fed human/chimpanzee diets, in liver.**
(XLS)

Table S2 **Summary of Gene Ontology analyses.**
(XLS)

Table S3 **Biological processes enriched among genes significantly differentially expressed between livers of mice fed human/chimpanzee diets.**
(XLS)

Table S4 **Biological processes enriched among genes showing diet effects** (**using an effect size cutoff**) **in livers of mice fed human/chimpanzee diets.**
(XLS)

Table S5 **References to the experimental studies used by TRANSFAC to describe the V$KROX_Q6 motif.**
(XLS)

Table S6 **Biological processes enriched among mouse-specific differentially expressed genes.**
(XLS)

Table S7 **Biological processes enriched among mouse-specific differentially expressed genes, using an effect size cutoff.**
(XLS)
