# Supplementary figures and images for: Mechanisms of Dietary Response in Mice and Primates: A Role for EGR1 in Regulating the Reaction to Human-Specific Nutritional Content
Source: PLoS One. 2012 Aug 24;7(8):e43915. doi: 10.1371/journal.pone.0043915 (PMC3427207; doi:10.1371/journal.pone.0043915)

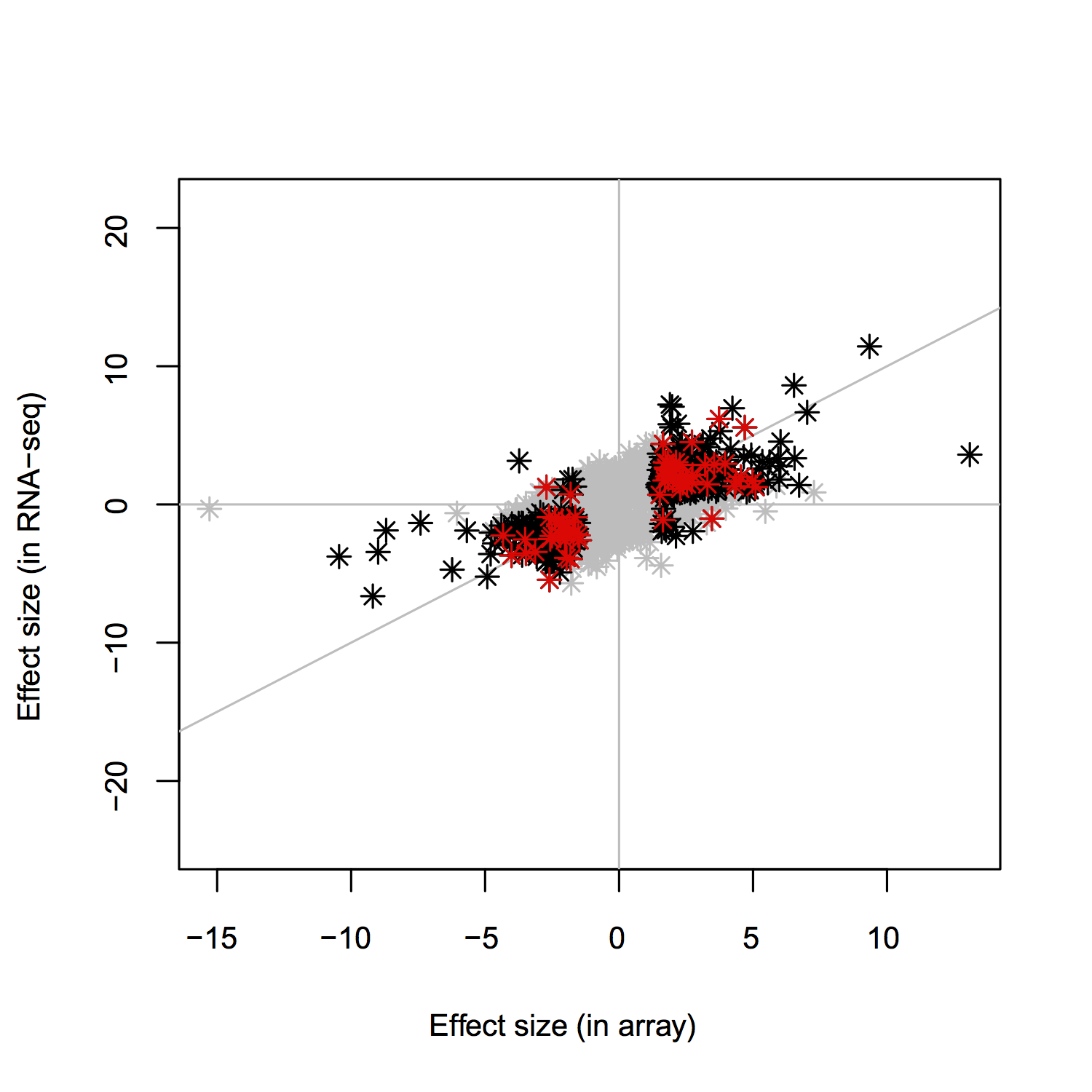

Supplement: Figure S1 — Consistency between microarray and RNA-sequencing datasets of primate liver gene expression. Scatter plot of effect sizes of differentially expressed genes (permutation based FDR <10%). Gray: genes differentially expressed in either primate dataset. Black: genes differentially expressed in both primate datasets. Red: genes differentially expressed in both primate datasets and in the mouse diet dataset. (TIFF) [file pone.0043915.s001.tiff]

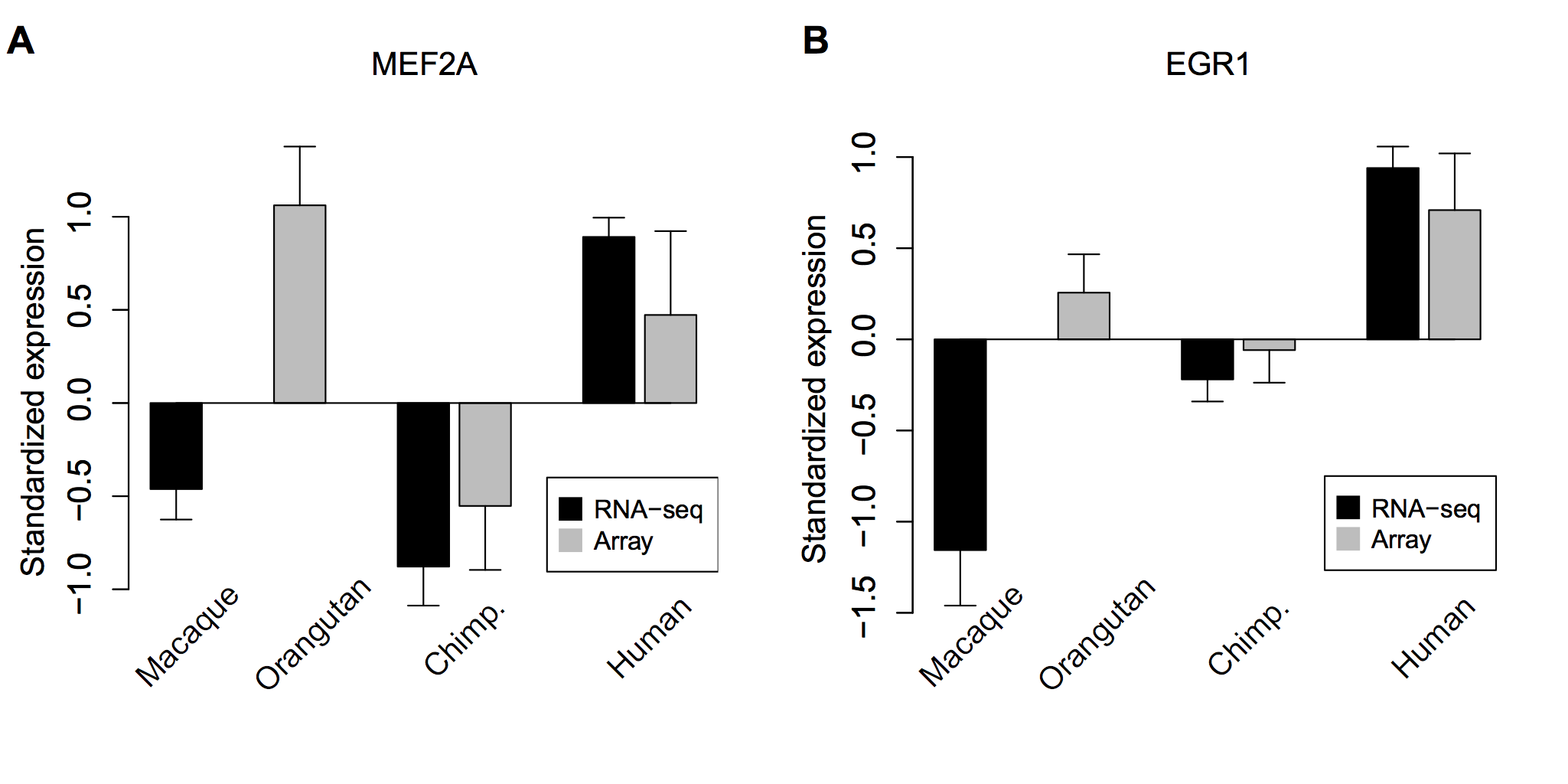

Supplement: Figure S2 — Liver expression profiles of MEF2A and EGR1 among primates. The two TFs, MEF2A (A) and EGR1 (B), were chosen for showing significantly non-random correlations with their predicted targets, where both the TFs and targets also showed species effects (|effect size|>0.8). Shown are relative expression levels (distances to mean levels in units of standard deviation across all liver samples) of the two genes in human, chimpanzee, orangutan and macaque samples, based on the microarray (‘Array’) and RNA-seq datasets. Results are expressed as mean ± SEM. EGR1 expression was significantly higher in humans compared to all other primates based on both the RNA-seq and the microarrays dataset (one-sided t-test p<0.05). (TIFF) [file pone.0043915.s002.tiff]

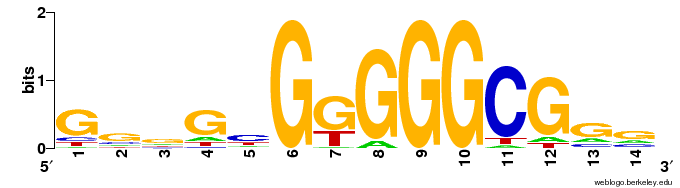

Supplement: Figure S3 — Sequence logo of V$KROX_Q6. This is the logo of V$KROX_Q6 created using WebLogo [70]. The sequence from the 6th to 10th position (GGGGG) is defined as the core element by TRANSFAC. Note that TRANSFAC arbitrarily chose sequences from the strand opposite to the one bound by EGR1 and uses a motif reverse complementary to this one (personal communication). (TIFF) [file pone.0043915.s003.tiff]

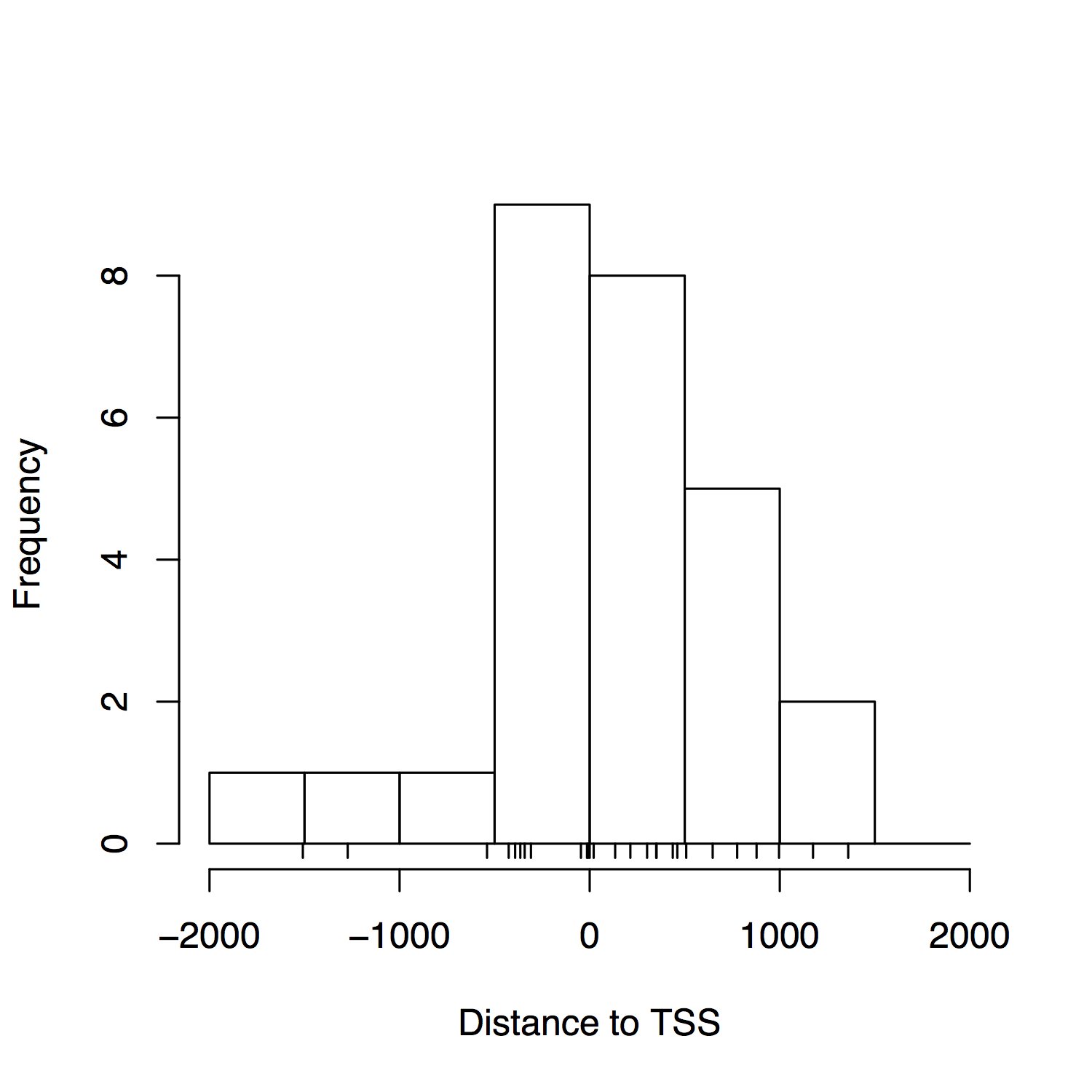

Supplement: Figure S4 — Positions of 27 binding sites of the 23 common genes. The x-axis is the distance to transcription start site (TSS). Positive values are downstream (3′ direction) to TSS and negative upstream. (TIFF) [file pone.0043915.s004.tiff]

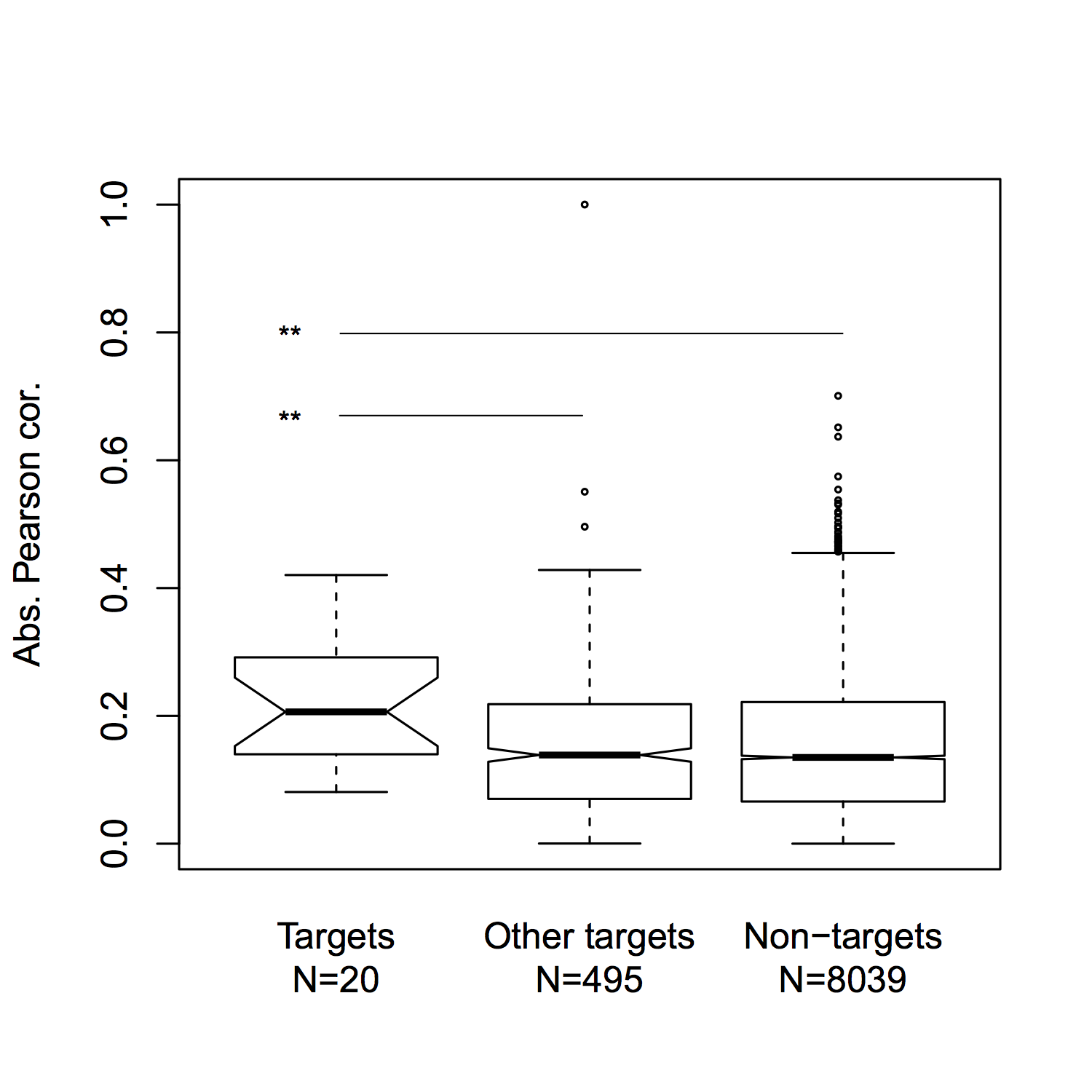

Supplement: Figure S5 — EGR1- target gene correlations in the human liver. The boxplots represent absolute value Pearson correlation coefficient distributions between EGR1 and three gene sets in a human liver dataset [36]. The gene sets are (a) the common EGR1 targets identified in the primate and mouse diet datasets (20 of the 23 genes expressed in the human liver dataset); (b) all predicted targets based on evolutionary conservation of the TFBS (n = 495); (c) all other expressed genes annotated as TF targets (n = 7,348). Asterisks indicate significance based on one-sided Wilcoxon tests, **: p<0.01. Note that we use absolute correlation coefficients because a number of observations suggest that EGR1 may act both as an activator and repressor of transcription. (TIFF) [file pone.0043915.s005.tiff]

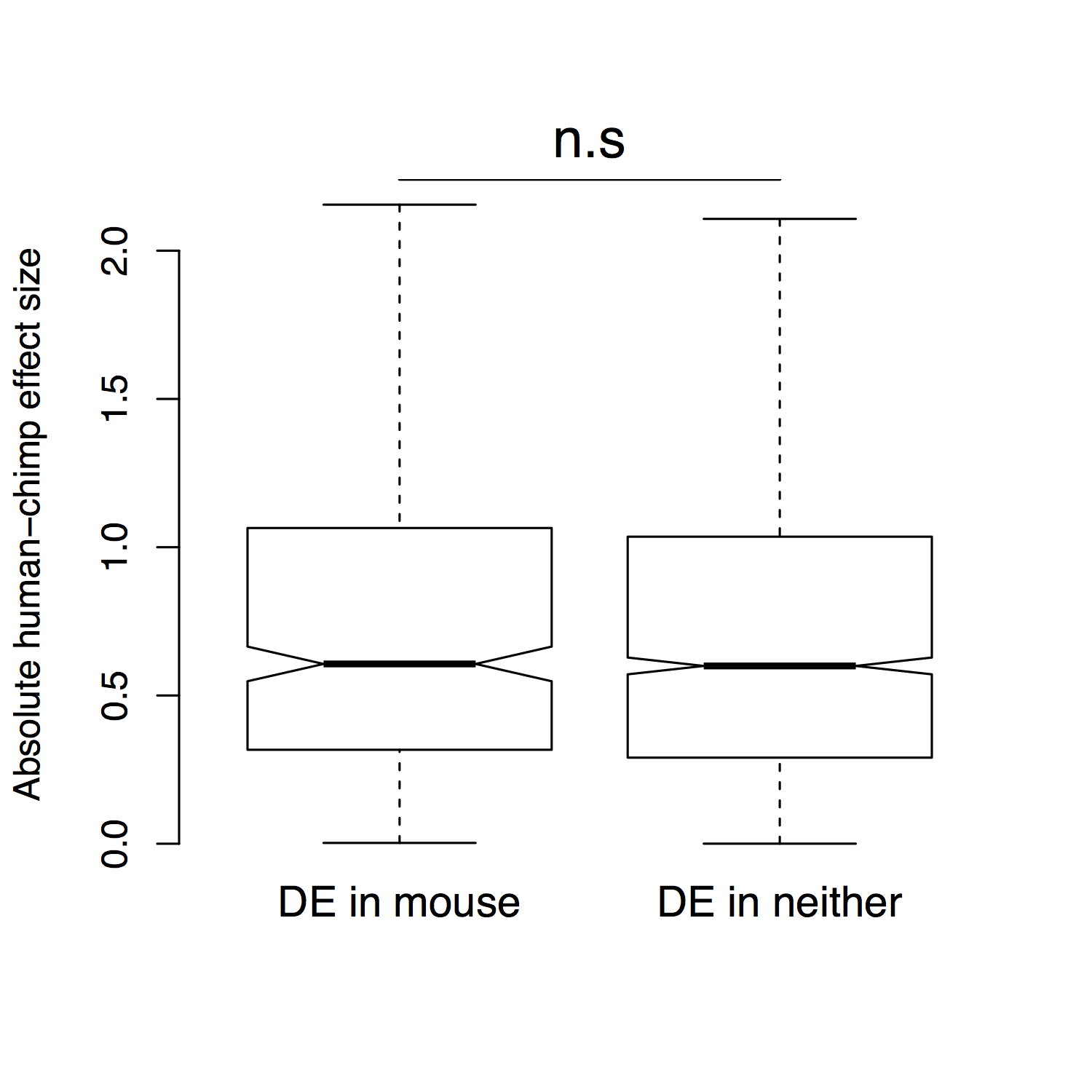

Supplement: Figure S6 — Mouse-specificity of dietary response is not due to lack of power. Boxplot of human vs. chimpanzee liver expression |effect sizes|, among genes showing or not showing diet effects in mice. “DE in mouse” indicates the 408 genes that were significantly differentially expressed (FDR <10%) only between mice fed human and chimpanzee diets but not between humans and chimpanzees in liver, “DE in neither”indicates the 1,725 genes that were differentially expressed neither between mice fed human and chimpanzee diets nor between humans and chimpanzees in liver. n.s: two-sided Wilcoxon test p-value>0.1. (TIFF) [file pone.0043915.s006.tiff]

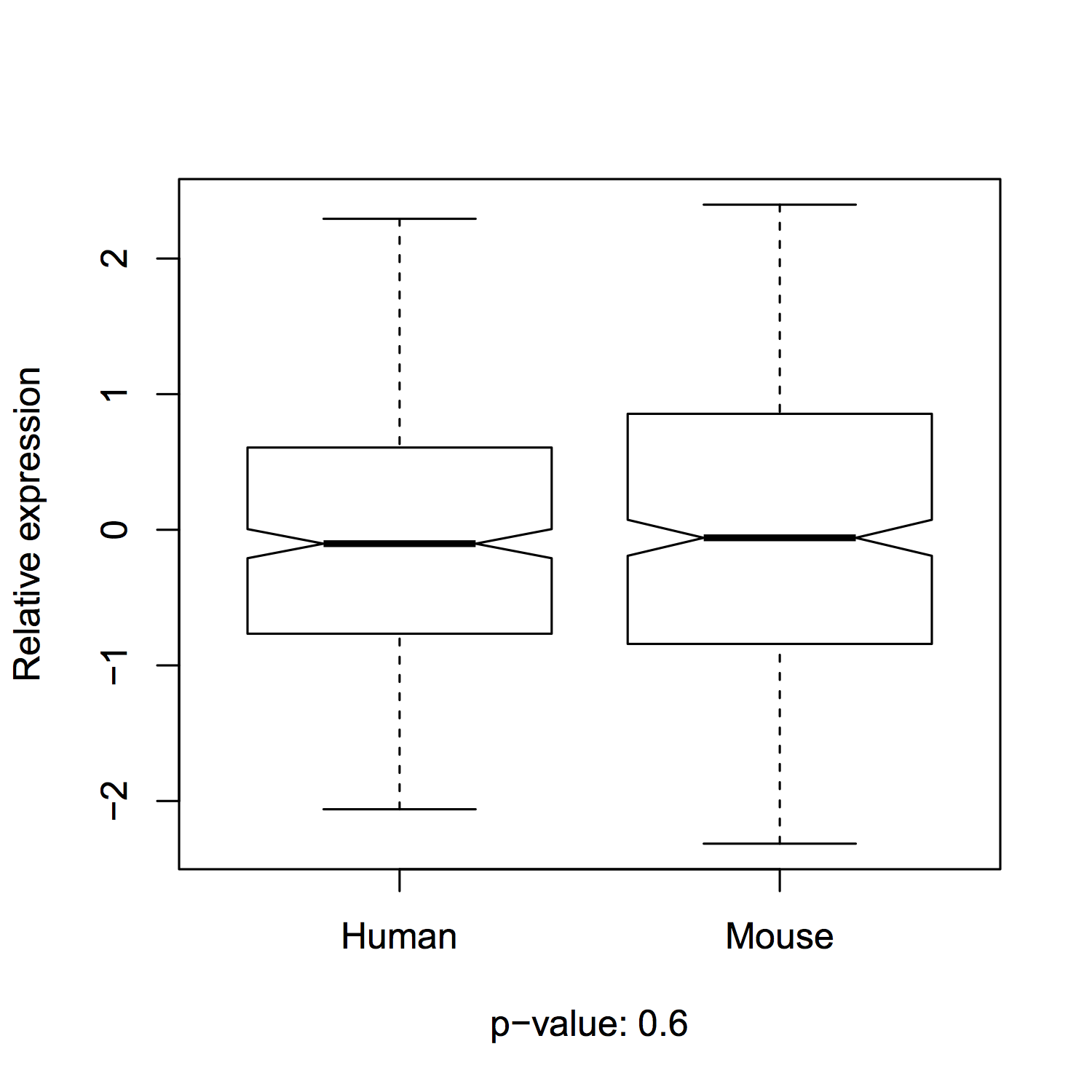

Supplement: Figure S7 — Mouse-specific genes show higher liver specificity but not higher expression levels in mouse liver. Boxplot of relative expression levels in mouse and in human livers (distance to the mean level in units of standard deviation across all expressed genes within liver), across mouse-specific differentially expressed genes (two-sided Wilcoxon p>0.1). (TIFF) [file pone.0043915.s007.tiff]

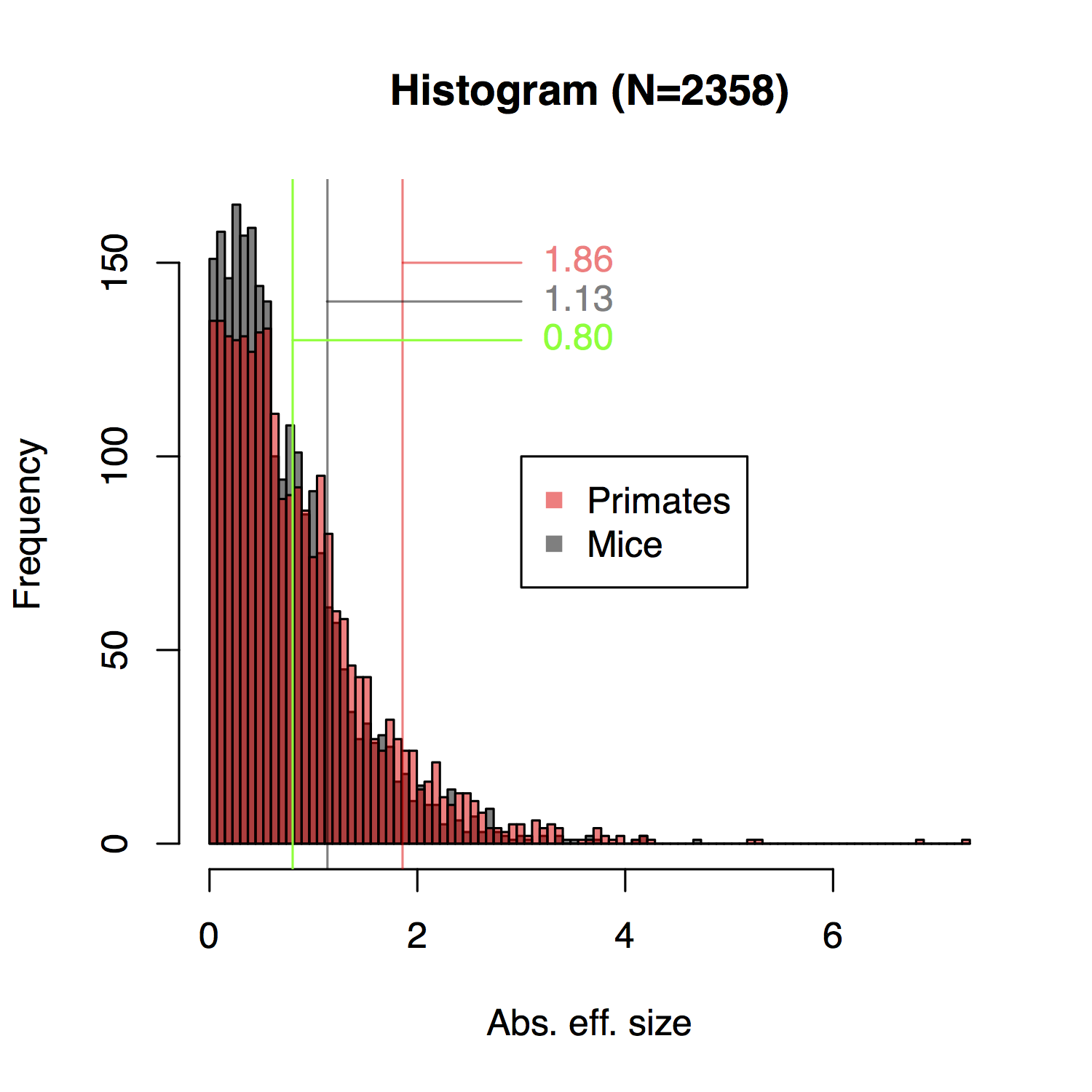

Supplement: Figure S8 — Distributions of absolute effect sizes of commonly detected genes. Shown are distributions of absolute effect sizes (|effect sizes|) of the 2,358 genes that were detected in both the primate dataset and the diet dataset. The gray and pink bars show the effect size at the FDR <10% cutoff in the mouse and at the cumulative FDR <5% in the combined primate datasets, respectively. The effect size cutoff 0.8, used in identifying regulatory effects in both datasets, is shown in green. (TIFF) [file pone.0043915.s008.tiff]

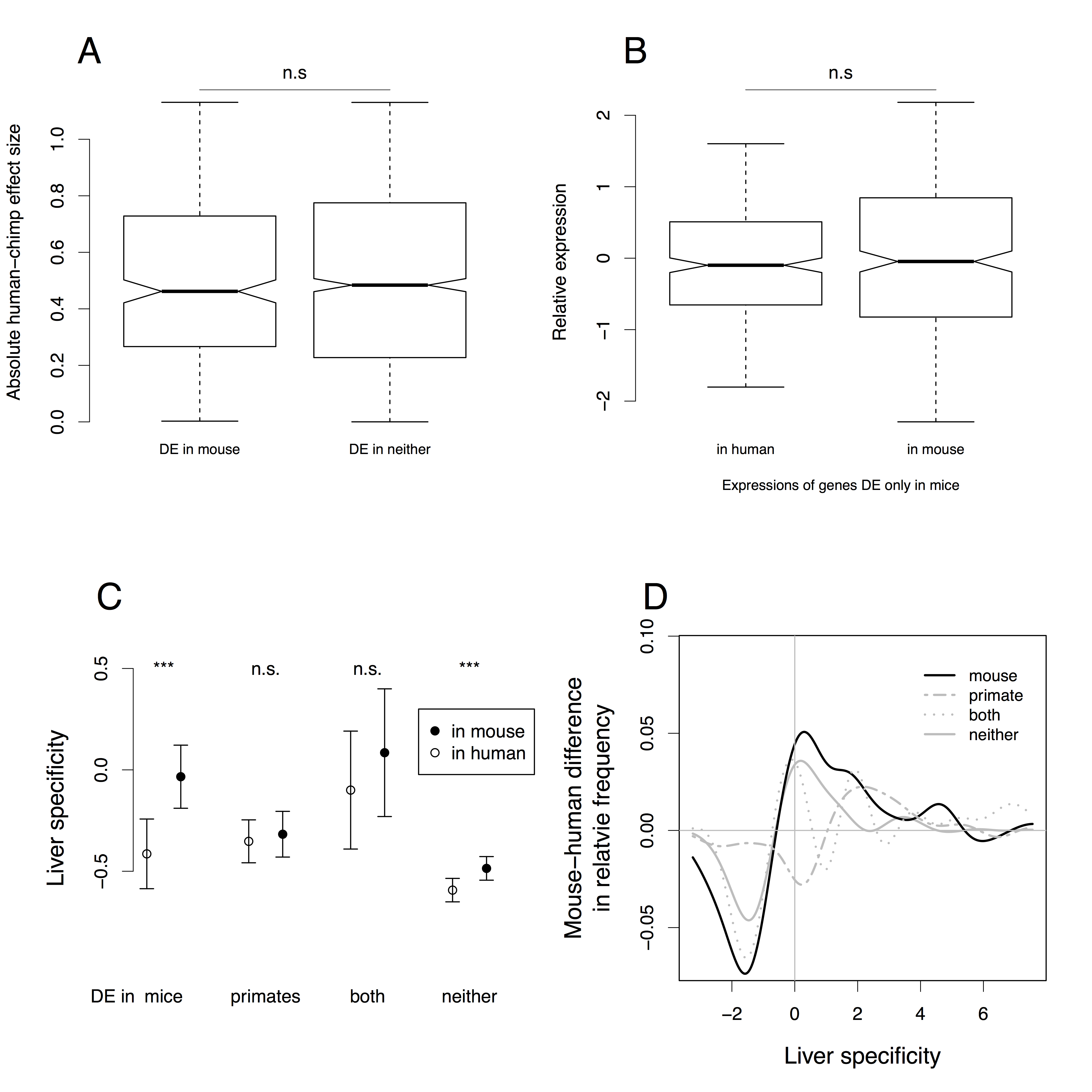

Supplement: Figure S9 — Mouse-specific dietary response under different cutoffs. To ensure that our results on mouse-specific dietary response were not affected by the choice of statistical cutoff, we repeated the analyses using an effect size cutoff (|effect size|>1.13) in the primate dataset equivalent to FDR <10% in the mouse diet dataset. (A) Boxplot of human vs. chimpanzee liver expression |effect size|, among genes showing or not showing diet effects in mice. “DE in mouse” indicates the 321 genes that were differentially expressed only between mice fed human and chimpanzee diets but not between human and chimpanzee in liver, “DE in neither” indicates the 1,392 genes that were differentially expressed neither between mice fed human and chimpanzee diets nor between human and chimpanzee in liver (see Figure S3). Here and in panel B–C, n.s: two-sided Wilcoxon test p>0.1; ***: p<0.001. (B) Boxplot of relative expression levels (distances to mean levels in units of standard deviation across all expressed genes within liver) of the 321 mouse-specific differentially expressed genes in human and mouse livers (two-sided Wilcoxon p = 0.47, see Figure S4B). (C–D) Liver-specificities of the 321 genes in human and mouse. Significance in all tests remains qualitatively similar to results shown in Figure 3. The difference in liver-specificities of mouse-specific differentially expressed genes between human and mouse after correcting human-mouse liver specificities of background genes is still significant (one-sided Wilcoxon test, p = 0.007). Compare to Figure 3. (TIFF) [file pone.0043915.s009.tiff]

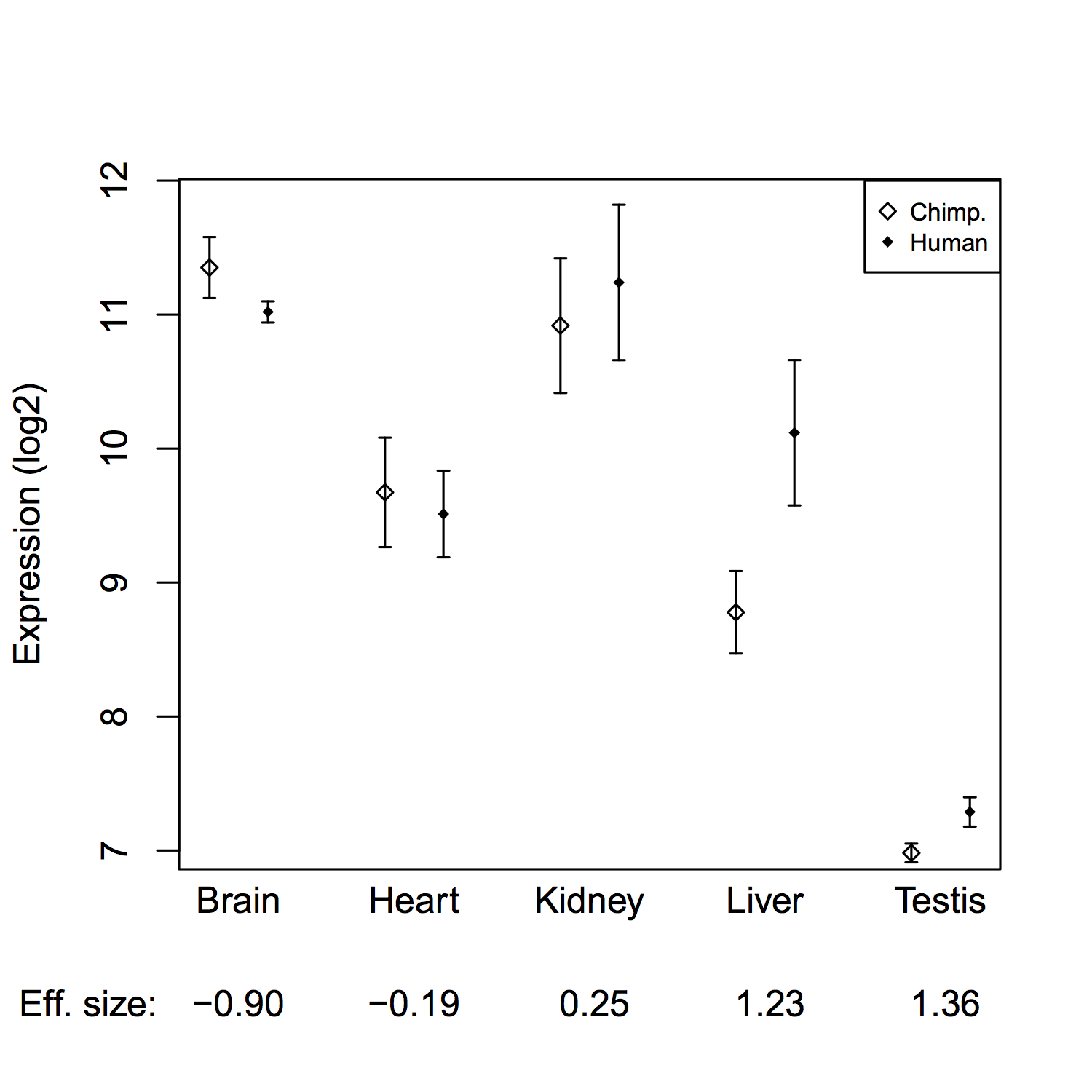

Supplement: Figure S10 — Expression divergence of EGR1 between human and chimpanzee in five tissues. Median expression of EGR1 in five tissues (brain, heart, kidney, liver and testis) [20]. The boxplots were plotted using the “boxplot” function in the R ‘graphics’ package [42]. Under default settings, the whisker ranges are calculated as: M±1.58×IQR/n0.5, where M, IQR and n are the median, interquantile range, and number of observations. Human-chimpanzee effect sizes are shown at the bottom line. (TIFF) [file pone.0043915.s010.tiff]

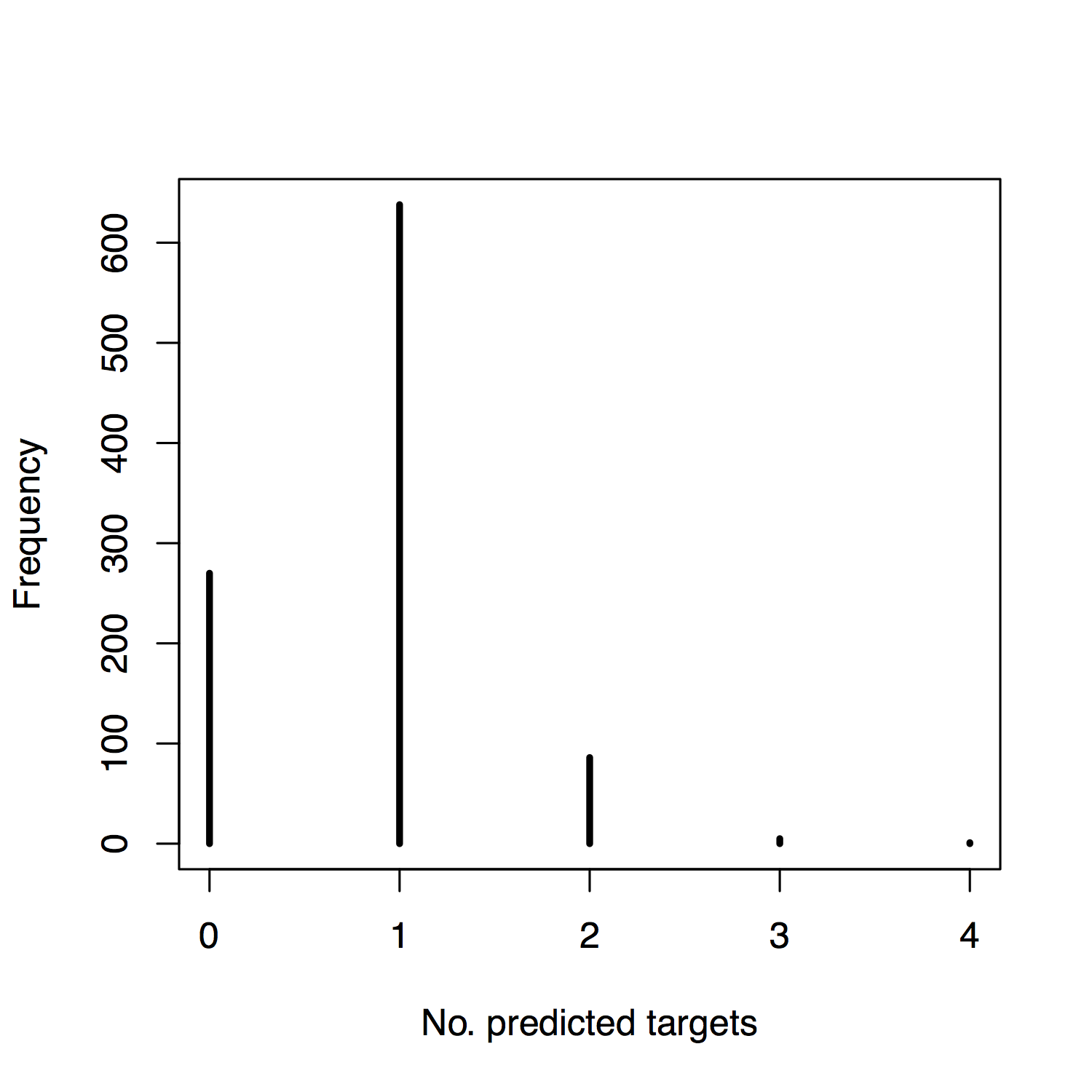

Supplement: Figure S11 — Distribution of numbers of predicted EGR1 targets based on random promoter sequences. Number of genes predicted as EGR1 targets based on random promoter region sequences shuffled maintaining dinucleotide levels (see Methods). The same criteria were used for the real promoter region sequences. The median of the distribution was found to be one. (TIFF) [file pone.0043915.s011.tiff]
